# Acute Myeloid Leukemia in Older Patients: From New Biological Insights to Targeted Therapies

**DOI:** 10.3390/curroncol31110490

**Published:** 2024-10-24

**Authors:** Pasquale Niscola, Valentina Gianfelici, Gianfranco Catalano, Marco Giovannini, Carla Mazzone, Nelida Ines Noguera, Paolo de Fabritiis

**Affiliations:** 1 Hematology Unit, S. Eugenio Hospital (ASL Roma 2), 00144 Rome, Italy; valentina.gianfelici@aslroma2.it (V.G.); marco.giovannini@aslroma2.it (M.G.); carla.mazzone@aslroma2.it (C.M.); paolo.de.fabritiis@uniroma2.it (P.d.F.); 2 Department of Biomedicine and Prevention, University of Rome Tor Vergata, 00133 Rome, Italy; gianfranco.catalano@uniroma2.it (G.C.); nelida.ines.noguera@uniroma2.it (N.I.N.); 3 Neurooncoemtology Units, Santa Lucia Foundation, I.R.C.C.S., 00143 Rome, Italy

**Keywords:** genomic profiling, targeted therapies, hypomethylating agents, venetoclax-based combinations, intensive chemotherapy, transplantation clinical trials, supportive care, quality of life

## Abstract

Acute myeloid leukemia (AML) is a heterogeneous blood-related neoplasm that predominantly afflicts older adults with a poor prognosis due to their physical condition and the presence of medical accompanying comorbidities, adverse biological disease features, and suitability for induction intensive chemotherapy and allogenic stem cells transplantation. Recent research into the molecular and biological factors contributing to disease development and progression has led to significant advancements in treatment approaches for older patients with AML. This review article discusses the latest biological and therapeutic developments that are transforming the management of AML in older adults.

## 1. Introduction

Acute myeloid leukemias (AMLs) are genetically diverse and heterogeneous age-related hematological malignancies [1,2,3]; the vast majority of patients are older and feature distinct biological and molecular backgrounds compared to younger individuals [2,3,4,5]. Indeed, there is a significant difference in the number and character of the disease’s drivers, with a more complex scenario of genomic abnormalities found in advanced age [4]. AML onset occurs in patients with a median age of 68 years [6], portraying a very dismal clinical outcome. Indeed, traditional treatment approaches, relying on intensive chemotherapy (ICT) regimens followed by allogeneic stem cell transplantation (SCT), produce a disappointing overall survival (OS) of about 10% [6]. Moreover, most patients are unsuitable to these standard measures due to the accompanying comorbidity burden, personal vulnerability, and socioenvironmental frailties. However, recent insights into disease pathobiology have improved our understanding of the driving biological mechanisms involved in AML development, clonal expansion, and disease progression [2,3,4,5]. The increasing knowledge of AML (genetic, epigenetic, metabolic, and microenvironmental) biology [7,8,9,10,11] is leading to a prominent role in a comprehensive diagnostic work-up in the prognostication process and tailored treatment decisions [1,8]. The most recent classification systems, such as the fifth update to the World Health Organization (WHO) Classification of Hematolymphoid Tumors (WHO-5) [12] and the International Consensus Classification (ICC) of myeloid neoplasms and acute leukemia [13], emphasize the significance of complex biological data over the relatively simplistic approaches used in the past [14,15,16,17]. After four decades of using one-for-all ICT, we are using targeted treatments tailored to single-patient disease biology and aimed at the specific vulnerability of leukemic stem cells (LSC). Targeting particular characteristics within tumor cell pathways is gaining traction, including focusing on genetic and epigenetic traits, apoptotic regulators, micro-environmental pathways, and immune-system modulators. Treatments based on these targets are currently available or in clinical development for AML [1,5,18]. All this effort and the availability of machine learning (ML) applications [19,20,21,22,23], with their transformative impact and potential perspectives for profound innovations, might change the fate of the vast majority of AML patients, including those older and frail [1,5]. All that makes these diseases a fascinating setting for clinical research and daily clinical practice. Hence, we summarized the most recent advances in biological understanding and therapeutic approaches in AML, excluding acute promyelocytic leukemia (APL), outside the aim of our review article.

## 2. Search Strategy and Selection Criteria

References for this updated review were identified through PubMed searches using multiple search terms related to several aspects of the biology, diagnosis, prognostication, and clinical management of older individuals with AML, considering only studies published in English until October 2024. With some exceptions, only papers published over the last three years have summarized the most recent developments as up-to-date as possible.

## 3. AML Profiling for Diagnosis and Prognosis

### 3.1. Nucleic Acids

**Figure 1:**
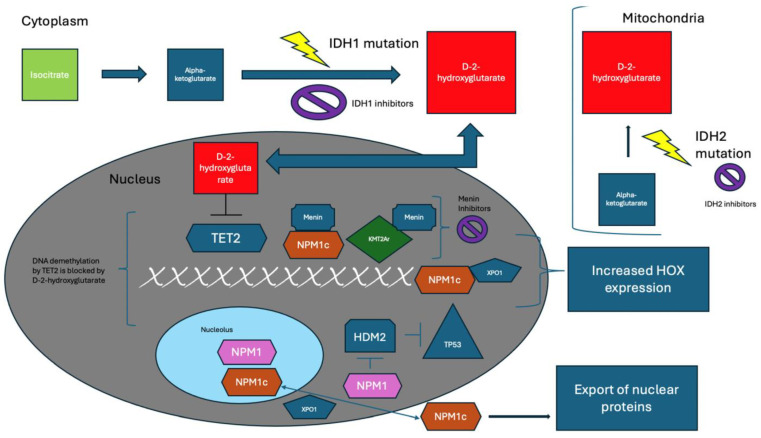
Effects of critical mutations on cellular function and pathophysiology of AML. In the cytoplasm, isocitrate is converted to alpha-ketoglutarate (A-KG). However, *IDH1* mutations reduce A-KG to D-2-hydroxyglutarate (D-2-HG), an oncometabolite. D-2-HG then travels to the nucleus and inhibits *TET2*, blocking DNA demethylation. Additionally, D-2-HG is created via reduction in the mitochondria by mutant *IDH2* enzymes from Krebs cycle-generated A-KG. *IDH1* inhibitors target the cytoplasmic reduction of A-KG to D-2-HG, while IDH2 inhibitors target the same process in the mitochondria. NPM1, which generally resides in the nucleolus and minimally binds XPO1, can travel to the nucleoplasm in stress conditions. In the nucleoplasm, it inhibits *HDM2*, which is significant because HDM2’s normal function is to inhibit *TP53*. Thus, by inhibiting *HDM2*, *NPM1* can increase *TP53*, which has important implications for cell regulation in stressful conditions. Mutant *NPM1* (NPM1c) has a higher affinity to *XPO1* and is prone to nuclear export, leading to critical protein export from the nucleus. Additionally, the consequent result of mutant NPM1 and *XPO1-NPM1c* can increase *HOX* expression. Furthermore, *NPM1c* and *KMT2Ar* interact with menin, facilitating leukemogenic cellular changes, which can be targeted via menin inhibition. Reprinted from Figure 1 in Ref. [3].

The development of molecular biology technologies represented a game changer in the knowledge of cancer biology. AMLs are pathologies of the hemopoietic stem cell (HSC), involving rapid growth and accumulation of abnormal leukemic cells in the bone marrow (BM) and peripheral blood (PB) as well as in extramedullary sites [1]. Phenotypic features reflect distinct backgrounds among HSC at different stages of differentiation, LSC, and proliferating AML blasts [2,16,18]. Understanding the molecular mechanisms underlying AML pathogenesis (Figure 1) [5] is essential for developing novel therapies [24,25,26,27,28,29,30] for specific biological targets [2,31]. Whenever possible, the comprehensive mapping of molecular alterations through a study of single alterations with frequent mutations [5,7,8] and next-generation sequencing (NGS) panels should be performed beyond conventional karyotyping [1,16,17]. In clinics, there are three main categories [1,5] of AML: so-called primary disease, such as de novo or newly diagnosed (ND) AML, secondary (s-AML), and therapy-related AML (t-AML) [1,5,32]. The distinction reflects fundamental biological underpinnings since de novo AMLs feature a less complex background than diseases emerging from previous hematologic malignancies [1,32], such as myeloproliferative disorders (MPNs) [32,33] and myelodysplastic neoplasms (MDS) [34] as well as from clonal hematopoiesis (CH), which is long-lasting premalignant conditions prevailing in individuals over 70 [35]. Also, t-AMLs that result from exposure to chemotherapeutic agents, radiation, or toxins [32] express a particularly complex biology. Accumulating acquired somatic mutations, interfering with the normal development of HSCs, drives the leukemic process. In this regard, CH-related genes (*ASXL1*, *TET2*, *SRSF2*, and *DNMT3A*) are most frequently mutated in older patients [2,32]. In most cases, they appear to be a relatively early event in leukemogenesis. In contrast, other genomic abnormalities, including mutations in *FLT3*, *NRAS*, and *RUNX1* [2,5,33], tend to be acquired later during leukemia development. As mentioned before, it is also important for therapeutic decisions to categorize acquired genetic mutations that characterize AMLs into specific pathways [2,5,32]. Activating mutations involving genes that sustain cell proliferation and survival, such as *FLT3*, *NRAS*, *KRAS*, and *c-KIT*, lead to the uncontrolled growth of LSCs. In contrast, mutations in genes related to DNA repair (e.g., *TP53*) [36,37,38], cohesin complex (e.g., *RAD21*), and spliceosome machinery (e.g., *SF3B1*, *SRSF2*, *U2AF1*, and *ZRSR2*) contribute to leukemogenesis and disease progression [2,5,33]. In addition, transcription factors (e.g., *CEBPA*, *RUNX1*, *PML/RARa*) and epigenetic regulators (e.g., *TET2*, *IDH1/2*, *DNMT3A*, *ASXL1*, and *EZH2*) affect genes involved in hematopoietic metabolism and differentiation [2,5]. Also, mutations in specific genes that control epigenetic status, such as DNA methylation and histone modifications, are crucial in the development of AML. These mutations lead to abnormal epigenetic patterns, changes in gene expression profiles, and the disruption of the differentiation of HSC [2,5,39]. In AML secondary to MPNs, the most common mutation is *JAK2 V617F*, which accounts for 98% of polycythemia vera (PV) and 55–60% of essential thrombocythemia (ET) and myelofibrosis cases [32]. However, it is important to thoroughly examine the genomic data to identify other potential candidates as leukemic driver mutations (oncosuppressors, epigenetic regulators, spliceosome modulators, or signal transduction genes). In contrast, in MDS, the most frequent mutations directly affect members of the spliceosome, such as *SF3B1*, *SRSF2*, *U2AF1*, and *ZRSR2*, as well as genes involved in DNA methylation and chromatin remodeling, such as *TET2*, *DNMT3A*, *IDH1/2*, and *ASXL1* [34]. A recently published study [40] reported on 49 PV and ET patients on the way to s-AML progression. The genetic sequencing demonstrated that cells might acquire so-called “long-term” (e.g., *TP53* and ATM) “short-term” mutations. In such a study, the former developed over many years and conferred a poor prognosis, whereas the latter led to rapid malignant transformation [40]. Finally, genetic variations in DNA excision repair systems, essential in maintaining genomic integrity and stability, can have a role in leukemogenesis [41]. As a result, the development of AML involves a complicated interaction of genetic mutations, epigenetic changes, and disrupted cellular signaling pathways. These factors lead to the transformation of normal HSCs into LSCs with uncontrolled self-renewal properties, driving the progression of the disease [2,3,4,5]. In myeloid-oriented LSCs, the phenotype CD34/CD123/CD25/CD99+ correlates with *FLT3-* internal tandem duplication (*ITD*)-positivity [42]. These cells overexpress CD99 antigen, which, due to its limited presence in normal cells, could be an optimal target for monoclonal antibody treatments or chimeric antigen receptor (CAR) T-cell therapy approaches [43,44]. *FLT3* mutations, such as *ITD* and tyrosine kinase domain (*TKD*), activate the *FLT-3* receptor pathway, leading to the uncontrolled growth of LSCs. *FLT3-ITD* allelic ratio (AR) plays a role in the context of *NPM1* mutations, where AR < 0.5 or ≥0.5 defines favorable or intermediate prognostic categories [16]. Patients harboring *FLT-3* mutations present with increased blasts in BM and PB at the AML onset and tend to have shorter progression-free survival (PFS) and OS. In particular, *FLT-3 ITD* mutations significantly affect the complexity of disease biology and prognosis [24,25,45,46,47]. Other genetic abnormalities are potential targets for specific drugs [47,48]. For example, mutations in the active sites of isocitrate dehydrogenase 1 (*IDH1*) and 2 (*IDH2*), which are reported at frequencies of 6–16% and 8–19%, respectively, are significant in AML [25,48]. Indeed, the latter enzymes convert isocitrate to α-ketoglutarate, producing nicotinamide adenine dinucleotide phosphate (*NADPH*). Therefore, these gene mutations reduce α-ketoglutarate and increase the *NADPH*-dependent conversion of α-ketoglutarate to 2-hydroxyglutarate (2-HG). In turn, the accumulation of 2-HG in cells competitively inhibits α-ketoglutarate-dependent processes as the function of the *TET2* enzyme, whose mutation afflicts about 20% of AML [25,48,49,50]. The latter enzyme converts 5-methyl cytosine to 5-hydroxy methylcytosine. Therefore, *IDH 1/2* or *TET2* mutations affect cytosine 5-hydroxymethylation of DNA, leading to mutually exclusive hypermethylation patterns [25,49,50]. Furthermore, high levels of 2-HG inhibit cytochrome C oxidase, making cells more susceptible to apoptosis when the activity of B-cell leukemia/lymphoma 2 (*BCL-2*) is inhibited [51]. For AMLs characterized by mutations in specific genes such as lysine methyltransferase 2a (*KMT2A*, also known as *MLL1*) [52] and nucleophosmin-1 (*NPM1*) [53,54], new compounds, such as menin inhibitors [55,56,57,58,59,60,61], have been developed and are currently in clinical investigations. *KMT2A*, located on chromosome 11q23, is a DNA-binding protein essential for regular cellular growth. The interaction with some proteins, such as menin, which regulates gene expression through histone methylation, influences its DNA binding. Abnormalities in the *KMT2A* gene occur in 70–80% of cases of infant leukemia, and they are rarely found in older AML patients [24,59,60]. Still, they are common in those with t-AML [32,58,61], particularly if they have received topoisomerase II inhibitors. The *NPM1* gene encodes a multifunctional protein prevalently located in the nucleoli and shuttles between the nuclear and cytoplasmic compartments. Importantly, *NPM1*-mutated AML cells exhibit abnormal cytoplasmic localization [53,54]. *NPM1* is a nuclear chaperone protein with several cellular functions, including ribosomal synthesis, stress response, and genomic stability [53,54,55]. In AML with normal karyotype, *NPM1* is the most commonly mutated gene (30–35% of adult and 50% of elder AMLs) [54,55]. Notably, overlapping features between t-AML and de novo *NPM1* AMLs suggest they can represent a single disease entity [62].

AML driven by the rearrangement of the mixed lineage leukemia-1 gene (*MLL1* or *KMT2Ar*) or mutation of the *NPM1* requires the chromatin adapter protein menin, encoded by the *MEN1* gene, to sustain aberrant leukemogenic gene expression programs [62,63]. Generally, *NPM1* mutations indicate a less severe prognosis without other genetic alterations, and the growth of *NPM1*-mutated AML is responsive to menin inhibitors [56,57,58,59,60,61]. Additionally, nucleoporin 98 (*NUP98*) [60], a gene located on chromosome 11p15, is involved in nuclear membrane transport and acts as a transcription factor in the *NPM1*. In approximately 1–2% of adult AML patients, *NUP98* fuses with one of over 30 different partners, contributing to the development of leukemia. *NUP98* fusions are usually associated with poor prognosis and may lead to resistance to chemotherapy [60]. Like *MLL* and *NPM1* mutations, *NUP98* fusion proteins bind to chromatin near homeobox (*HOX*) genes, causing their overexpression through various mechanisms, including altered DNA methylation and acetylation. The binding of these fusion genes to chromatin depends on both *MLL* and menin [63]. In preclinical studies, leukemic cells with *NUP98* fusions responded to menin inhibition [55,56,57,58,59,60,61]. Lastly, *HOXA9*, a transcription factor overexpressed in approximately 70% of AML and associated with poor prognosis, increased chemoresistance, and higher relapse rates, can be successfully inhibited in in vitro human AML models [64].

### 3.2. Proteomic Profiling

Genetic and transcription data are seldom exhaustive in describing cellular biomechanics: proteomic analyses, single protein levels, and specific phosphosites must be considered to identify clinically relevant diagnostic and predictive patterns compared to transcriptomics or genomics alone. The overexpression of the *BCL-2* protein family, located in the cytoplasm, can promote cell survival control by capturing proapoptotic proteins. Thus, it prevents mitochondrial membrane permeabilization and cytochrome C release, which activates apoptosomes [65]. So, *BCL-2* is crucial for the survival and growth of leukemic cells [65,66]. Interestingly, AML patients have a wide range of antiapoptotic and proliferation index due to differences in the maturation stage and severity of the disease at diagnosis. A recent study using double-labeling and mutually exclusive *Ki-67* and *BCL-2* dynamic markers demonstrated that AML blast cells simultaneously showed increased antiapoptotic and reduced proliferative marker expression [66]. Specific compounds, such as venetoclax [24,67,68], can target these abnormal biological features. In our previously reported experience, we demonstrated that in neoplastic cells only, myeloid cell leukemia 1 (*MCL1*) [18] directly binds to Hexokinase 2, forming a complex with mitochondrial voltage-dependent anion channels (VDACs, mitochondrial porin) on the outer mitochondrial membrane [69], protecting this complex the *MCL1* protein from degradation. Finding a way to disrupt this complex through early *MCL1* inhibition might help rescue high-risk AML patients. Furthermore, several researchers have shown that venetoclax’s resistance to LSCs depends on oxidative phosphorylation (*OXPHOS*) and that adding the inhibition of fatty acid oxidation [70] or *MCL1* in the context of monocytic AML restores sensitivity to this agent [71]. Profiling bioactive molecules such as sphingolipids can be a predictive tool for AML. These molecules have different activities in regulating cell proliferation, differentiation, apoptosis, and immune cell activation and have implications for AML pathogenesis and therapeutic resistance [72]. In this regard, a study on the molecular landscape (genomic, transcriptomic, and proteomic) provided insights into ex vivo drug response in 210 patients with AML, allowing the identification of four proteogenomic subtypes and specific drug response patterns [73].

### 3.3. Metabolic Profiling

Leukemogenesis alters the way how LSCs consume and process nutrients in a flexible and adaptable manner [10,74,75]. In developing AML and its response to treatment, metabolic reprogramming plays a crucial role. At the outset, the specific cellular metabolism of AML could lead to the development of new stratification systems and potentially novel targeted therapies. Phenotypic features indicate distinct metabolic backgrounds among HSCs at different stages of differentiation, such as LSCs and proliferating AML blasts [2,5,10,76,77]. The differential regulatory functions of mitochondria between normal HSC and LSC play a fundamental role in the response to oxidative stress [78,79,80]. Due to the central role of glycolysis, glucose uptake and mitochondrial function in malignant cell dynamics are essential in leukemic metabolism, particularly in maintaining mitochondrial physiological state and stability [74]. Moreover, leukemic cells utilize various metabolic pathways simultaneously, including glucose derivatives, lipids, amino acids, and *OXPHOS* for energy production. They also protect mitochondrial membrane permeabilization through a proton leak equilibrating effect. The abnormal overexpression of *MCL1*, *BCL2*, and related antiapoptotic *BH3* family genes helps to stabilize mitochondria, promoting tumorigenesis and drug resistance in various cancers [81,82]. Therefore, it may be crucial to identify differences in sensitivity to mitochondria-targeting therapies. In this context, proton leak values identify two distinct populations with different prognoses, considering that previously reported metabolic profiling intercepts different cellular features already known to be important in leukemic cells [18,83]. In particular, a high proton leak is associated with *FLT3* mutations, even though its impact on prognosis appears independent [18]. Therefore, integrating information on the leukemia-specific cellular dependencies in regulatory pathways or metabolism at onset could lead to developing a new stratification system and therapies targeted at specific alterations.

### 3.4. Ambience Profiling

The role of the BM microenvironment in AML development and progression is the focus of several active investigations [84,85,86]. With this regard, an emerging understanding of the role of tumoral angiogenesis points out the impact of intercellular signaling and endothelial cell subsets in shaping HSC homeostasis in the BM niche [87]. LSCs depend on survival and expansion from the same protective network as normal HSCs; at the same time, LCSs’ intrinsic characteristics allow for successful competition and substitution of normal HSCs in the microenvironmental niches [86,87]. It is relevant to note that AML cells can use different strategies to avoid ferroptosis cell death, controlled by three main cellular processes: oxidative stress, iron, and lipid metabolism [88]. Bian Y et al. demonstrated that high methionine uptake in leukemic cells can induce the epigenetic reprogramming of antitumor T cells and impaired antitumor function [89]. While directly competing with immune cells for essential nutrients, tumor cells’ metabolism by-products, lactic acid, reactive oxygen species [90], kynurenine, polyamines, adenosine, and cholesterol shape the BM microenvironment in an immunosuppressive fashion [84,86,87,91,92]. An altered inflamed and immunosuppressive BM microenvironment supports LSCs’ survival and proliferation [86,87,88,93,94,95,96,97]. In addition, the dysregulated interactions of LSCc with stromal cells and altered cytokine signaling lead to disease progression and therapy resistance [86,87,88]. Recently, Lasry et al. proposed that the immune microenvironment and the features of its inflammatory state can allow for AML classification. Indeed, they identify immune populations uniquely expanded in AML patients with high phlogistic features, describing an inflammation-related gene signature (iScore) with independent prognostic impact in AML [97].

### 3.5. Extensive Data Analysis, Machine Learning, and AI Tools

ML and artificial intelligence (AI) enable the analysis of large amounts of data obtained through various methods. Ongoing efforts can effectively diagnose and comprehensively characterize AML beyond simply identifying blast cells with their abnormal features, as it combines multiple analyses [19,20,21,22,23]. The use of these technologies shows promise in providing significant added value and improving the management of the diverse biological data associated with AML [7,8,9,16,17,98,99]. A study by Makishima et al. evaluated the mutation landscape in 2250 MDS patients that evolved to sAML. They identified two groups: one with mutations in *FLT3*, *PTPN11*, *WT1*, *IDH1*, *NPM1*, *IDH2*, and *NRAS*, which conferred a lower risk of progressive disease, and the other with mutations in *TP53*, *GATA2*, *KRAS*, *RUNX1*, *STAG2*, *ASXL1*, *ZRSR2*, and *TET2*, which were predictive of a higher risk of aggressive AML evolution [99]. In 2020, Warnat-Herresthal et al. examined transcriptomic and genomic data from more than 100 studies and consistently detected AML in a near-automated, low-cost method [100]. In a retrospective survey of 241 patients, Cheng et al. demonstrated that a trained AI program could identify patients with AML and tell apart physiological cells based on multiparametric flow cytometry (MFC) [101]. Automated analysis results identify genes with survival differences in AML; indeed, *DNM1*, *MEIS1*, and *SUSD3* are potential risk factors in AML, significantly associated with AML subtypes [101]. Additionally, a recent study found a link between the *FLT3-ITD* mutation and an abundance of progenitor-like cells. AML cells showed disrupted gene expression patterns, with a combination of genes related to LSCs and myeloid development. These findings have prognostic implications [102]. A similar approach could identify potentially resistant cell clones, especially against anti-*FLT3* inhibitors, from the onset sample of the disease [103]. Therefore, using single-cell sequencing and AI (machine learning classifier), researchers can identify different LSCs that may produce treatment-resistant cell clones within the same tumor. Those are a few examples of the growing mass of studies coming out daily; the field is up-and-coming, but more time and specific expertise are needed to ripen it.

#### 3.5.1. Diagnostic and Classification Challenges

In a real-life scenario, conducting a thorough work-up for all patients, including fit young adults and older patients, is recommended regardless of whether they are candidates for ICT and allogeneic SCT. The initial clinical work-up should allow for the accurate diagnosis, precise classification, and adequate risk stratification of AML patients by integrating clinical history, patient fitness, BM and PB morphologic examinations, MFC [104], cytogenetic findings, NGS, and metabolic profiling. NGS and MFC diagnostic tests accommodate new classification schemes (Table 1, Table 2 and Table 3) and provide precise treatment indications [16]. The previous AML classification by the WHO (fourth edition, WHO-4) [105] used blast counts equal to or higher than 20% in the BM/PB as the criterion for AML diagnosis. However, there were exceptions for APL and core binding factor (*CBF*) AML (i.e., *RUNX1*-*RUNX1T1*, *CBFB*-*MYH11*), which allowed for the AML diagnosis with a blast count of less than 20% without a specific cut-off value [105]. The WHO-5 classification contains some differences from the previous edition. The WHO-5 [12] distinguishes AML based on differentiation features from those genetically defined (Table 1 and Table 2). In this updated classification, the requirement of 20% blasts is not necessary for diagnosing AML in the presence of specific genetic abnormalities (Table 1), except for AML with *BCR-ABL1* fusion, AML with *CEBPA* mutation, and those harboring other rare genetic alterations, which still require 20% blasts. Studies have shown that patients with any of these abnormalities and less than 20% blasts, previously classified as per WHO-4 [105], have clinical outcomes similar to those with higher blast counts. One notable change is the inclusion of a new category by ICC classification for patients with a blast percentage of 10–19% as a separate entity of MDS/AML (Table 1 and Table 3), allowing these patients to participate in MDS or AML clinical trials and treated in clinical practice as the same clinical entities [13]. In contrast, the WHO-5 classification recognizes this entity as MDS with increased blast II. Additionally, both classifications recognize blastic plasmacytoid dendritic neoplasms (BPDN), a rare myeloid neoplasm, among AML. Notably, the ICC classification introduced a new hierarchical classification for AML in 2022, emphasizing the crucial role of genomic and molecular characteristics in determining treatment and prognosis. Moreover, the ICC classification emphasizes specific molecular abnormalities by introducing a separate entity for AML with *TP53* mutations, while the WHO-5 classification does not make this distinction. Additionally, the ICC focuses on specific genetic abnormalities that significantly impact disease classification, leading to substantial changes, such as categorizing therapy-related AML as a diagnostic qualifier rather than a separate disease entity. Again, the WHO has introduced a new category called myeloid neoplasms post-cytotoxic therapy. Table 3 shows the critical differences between the two new classifications. These discordances include changes in the percentage of AML cases defined purely by morphology, an increase in AML cases related to MDS, and a shift in the largest group to other genetically defined AMLs [12,13]. With this regard, a recently reported study evaluated 860 patients with a diagnosis according to the previous WHO classification and found that the 2022 AML classifications significantly improved diagnostic schemes [106], intending to facilitate the application of novel and innovative agents tailored to biological and molecular targets [107,108]. Therefore, developing a unified model to standardize treatment approaches and enrollment in clinical trials for AML, based on the similarities between the WHO-5 and ICC diagnostic schemes, would be beneficial [106,108]. Indeed, both classifications emphasize genetics-based definitions with similar basic concepts and a significant degree of agreement despite some areas of incomparability, such as *TP53* mutant AML [20,36,37,38], which still need to be addressed.

#### 3.5.2. Older Patients: New Approaches in Risk Assessment and Monitoring of AML

The recent development of innovative treatment approaches for older AML patients, including novel and effective tailored agents targeting biological and molecular factors, has made the prognosis and the monitoring of therapeutic responses increasingly important in decision-making and clinical management [109,110,111,112,113,114]. The current estimation of prognosis for AML is complex and depends on patient-related factors, AML manifestations at diagnosis, and disease genetics [16,115]. While NGS platforms have improved our understanding of some molecular aspects of AML biology, prognostic factors identified in patients treated with ICT are becoming less reliable as new insights emerge and novel effective treatments become available. Currently, the most commonly used consensus risk stratification guidelines for AML are those from the European Leukemia Net (ELN) [16] (Table 4) and the National Comprehensive Cancer Network (NCCN) [17]. Real-life data of 624 ND AML patients from 1998 to 2014 [116] and deep learning technologies in older patients [23] have validated the ELN genetic risk stratification [16]. Genes associated with adverse prognosis now include *TP53*, *ASXL1*, *BCOR*, *EZH2*, *RUNX1*, *SF3B1*, *SRSF2*, *STAG2*, *U2AF1*, or *ZRSR2*. Furthermore, in line with the ICC 2022 classification, in-frame mutations affecting the *bZIP* region of *CEBPA* are now categorized as the favorable-risk group (Table 4) [13], replacing biallelic *CEBPA* mutations [12,13]. However, these guidelines have been developed considering the fit of younger patients submitted to ICT [16]. Therefore, they have some limitations, particularly for patients with advanced age and those with frailties and comorbidities, for which it is unclear whether this classification applies to adults aged 60 and older who receive lower-intensity treatments [115]. Therefore, as the trend towards using lower intensive treatments for most elderly patients continues [67,117,118,119], active research in this field has provided new tools for prognostication for older AML treated with less intensive regimens [120,121,122]. A recent study investigated how the ELN risk affects the prognosis of ND AML patients aged 60 and older who are given lower intensive therapy and refined the risk stratification for this group [120]. The study included 595 patients, with 11% having favorable risk, 11% having intermediate risk, and 78% having adverse risk based on the ELN AML criteria. Therefore, ELN risk was a reliable prognostic factor for OS but did not differentiate between favorable and intermediate AML risk. A multivariable analysis that included 316 patients found that the *IDH2* mutation was an independent favorable prognostic factor, while the *KRAS*, *MLL2*, and *TP53* mutations were associated with an unfavorable outcome. Based on these findings, a “mutation score” was calculated for each combination of these mutations. Therefore, adverse-risk patients encompass two distinct categories: those with −1 to 0 points (“Beat-AML-intermediate”) and those with one or more points (“Beat-AML-adverse”) [120]. After conducting analyses, the authors developed a refined risk classification for older ND AML patients. They combined the ELN favorable- and intermediate-risk groups into a newly defined “Beat-AML-favorable-risk” category and included the mutation scoring within the ELN adverse risk. This approach proposed refined Beat-AML-favorable- (22%), Beat-AML-intermediate- (41%), and Beat-AML-adverse-risk (37%) groups, which showed improved discrimination for OS [120] compared to ELN AML categories [16]. In addition, investigating molecular signatures found in 159 AML patients treated with HMA/VEN allowed for a new prognostic system, such as the molecular prognostic risk signature (mPRS) [121]. The authors categorized patients into favorable, intermediate (*N/KRAS* or *FLT3*-internal tandem duplication mutations), and lower (*TP53* mutations) benefit groups. The OS rate for the entire cohort was 71%, ranging from 86% to 54% and 59% in the higher, intermediate, and lower-benefit groups. Moreover, the median OS and EFS also varied significantly, with the higher-benefit group having a median OS of 30 months and EFS of 19 months, compared to a median OS of 12 and 5 months and an EFS of 8 and 4 months recorded in the intermediate and lower-benefit groups, respectively [121]. Therefore, in this study, the mPRS classification accurately segregated groups of patients with AML treated with HMA/VEN, having been *N/KRAS* and *TP53*, the mutations that negatively affected outcomes [122]. Therefore, a new prognostic system for AML patients treated with azacytidine/venetoclax (AZA/VEN) (Table 5) has been proposed using emerging genetic data, on which the 2024 ELN recommendation for the setting of lower intensive therapy in the AML setting was based [122]. Based on *TP53*, *FLT3-ITD*, *NRAS*, and *KRAS* mutational status, this system distinguished patients into higher, intermediate, and lower benefit groups with different median OS outcomes. Specifically, 52% of patients fall into the higher-benefit group, 25% into the intermediate-benefit group, and 23% into the lower-benefit group [122]. Another important issue of growing importance in the setting of older AML patients receiving less intensive treatment is MRD detection, which is currently not a routine practice for older patients despite being a valuable guide for potential personalized medicine applications in lower-intensive treatments [109,110,111,112].

#### 3.5.3. A Comprehensive Approach in Older AML Patients

Therefore, the prognosis for an individual AML patient relies on both clinical features and the leukemic blasts’ immunophenotypic and cytogenetic/molecular characteristics. Patient age, comorbid conditions, and prior history also contribute to clinical manifestations and treatment. Notably, adverse risk factors are more common in older adults with AML. This finding leads to a generally inferior prognosis compared to younger patients. Indeed, while curative therapy for AML has traditionally relied on ICT and allogeneic SCT, these measures are associated with severe complications in older AML patients. The search for a reliable basis of data to discern responding individuals before submitting elderly patients to challenging therapies with no reasonable expectations of a positive outcome seems mandatory. Given the frequent complex comorbidity profile of the large majority of AML patients, we should pursue therapies with a low toxicity profile and the means to individuate single patient profiles significant to the tailored therapeutic choice. Therefore, despite remarkable advances in this field, novel risk stratification systems are essential in older patients with AML candidates to receive lower-intensity treatments by tailored agents and new effective combinations, facilitating new possibilities in this challenging setting [25,117,118,119,123].

## 4. Clinical Management of AML in Older Patients in the Current Era

**Figure 2:**
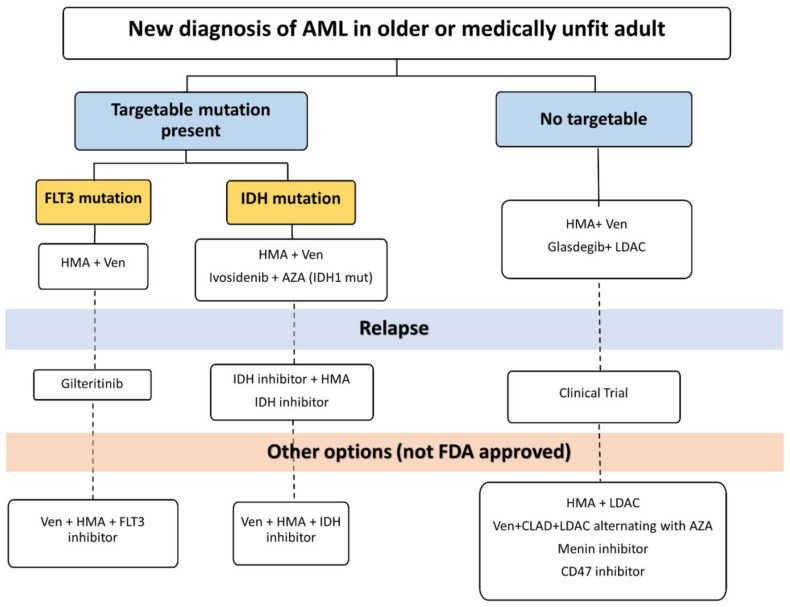
Suggested treatment algorithm for newly diagnosed AML. HMA: hypomethylating agent. AZA: azacitidine. LDAC: low-dose ara-C. CLAD: cladribine. Ven: venetoclax (adapted from [25]).

Treatment options and approaches for older AML patients have expanded significantly in recent years [16,17], in line with advancements in diagnostic technologies [7,8,9] and our understanding of the complex biological mechanisms [2,3,4,5] underlying these aggressive blood cancers [1]. Figure 2 [25] and Table 6 show the updated treatment algorithm and the currently available agents [24,25,26,27,28,29,30,31,32] for older AML patients, respectively. Additionally, Table 7 shows the ongoing clinical trials involving older AML patients.

### 4.1. The AZA/VEN “Revolution”

Before 2017, older AML patients who were deemed fit typically received standard ICT, while unfit patients aged 75 years and older were treated with a hypomethylating (HMA) compound as a single agent [124]. The outcome of AML in older patients relies on several factors, including age, overall health, and fitness status [115]. Moreover, managing AML in older patients is challenging due to decreased functional abilities and individual fitness [6,27]. As a result, most older patients with AML are not suitable for ICT, are more likely to have genetic features with negative prognostic impact, and may be resistant to treatments [1,2,5,25]. Over the last few years, efforts have allowed for the development of practical tools to determine which patients are fit or unfit for ICT or non-ICT in clinical practice [1,115,119], given expanded treatment options for older patients, including targeted and lower-intensity therapies [24,25,26,27,28,29,30,31,32] in both frontline and relapsed/refractory (R/R) settings [125]. In particular, the therapeutic association of venetoclax with HMAs, mainly azacytidine in the AZA/VEN combination, currently represents the standard of care adopted for older AML and ICT-unfit patients [1,123]. The primarily known “Ferrara criteria” [126] have been validated in this regard by a retrospective study in the ICT setting [127]. In this regard, a recent Italian retrospective survey suggested modifications of fitness criteria to accommodate the use of HMA/VEN, representing the treatment of more than one-third of AML patients compared to 2008–2016 [119]. The same study reported that the use of ICT decreased from 40% to 18%, and the use of HMA alone reduced from 19% to 13%. Thereby, there are suggestions to update the fitness criteria for patient candidates for HMA/VEN combination treatment due to the unique toxicity profile of this combination, which can lead to prolonged neutropenia and increased risk of infections [128,129,130,131]. The proposed updated fitness criteria include an age limit of 80–85, cardiac function > 40%, the absence of certain lung conditions, and the presence of an adequate caregiver [119]. Therefore, new therapeutic combinations incorporating venetoclax, which induces apoptosis in AML cells by *BCL-2* inhibition, have changed this treatment landscape [30,67,128,129,130,131]. The phase 3 VIALE-A trial included 431 patients 75 years older or with significant comorbidities. Patients were randomly assigned to receive the AZA/VEN combination or azacytidine alone [67]. Combining AZA/VEN resulted in a higher composite CR rate of 66% and OS of 14.7 months compared to 28% and 9.7 months for those treated with azacytidine alone [67]. Moreover, the durable efficacy and the maintained safety of AZA/VEN were confirmed at 43.2 months of median follow-up, with the reported OS of 14.7 months by this combination compared to 9.6 months by azacytidine alone [128]. A recently published meta-analysis of nine studies, including 1232 patients, confirmed a significantly higher composite CR rate and longer OS in older patients with ND AML treated with AZA/VEN compared to those who have received azacytidine monotherapy [129]. The former group of patients presented more severe neutropenia and gastrointestinal toxicity in comparison to those treated with azacytidine alone [130]. It is worth noting that in the ND NPM1-mutant AML setting, the combination of HMA/VEN achieves long-lasting remissions, as reported by a recent multicenter study involving 221 participants (treated with 147 ICT, 74 HMA/VEN) and comparing this combination to standard induction ICT [132]. The study’s authors found similar CR rates (ICT:85% vs. HMA/VEN:74%) in both groups. Importantly, in multivariate analyses, the two groups showed no differences in OS or the proportion of patients among those aged 60–75 years who proceeded to allogeneic SCT [132]. In the same setting of *NPM1*-mutant AML, the combination of HMA/VEN allowed for an effective bridge-to-transplant strategy for patients with molecular failure [133]. Of note, this treatment combination allowed for long-term efficacy in the challenging setting of extramedullary AML [134]. However, this treatment can lead to significant myelosuppression and complications, making it difficult for very old patients. However, in a recent study including 154 AML patients older than 80, the combination of HMA/VEN (azacytidine: 83% of patients) achieved a composite CR (cCR) rate of 73%. Additionally, the median OS for the entire cohort of patients and those who obtained a cCR was 8.1 months and 13.2 months of patients, respectively. Therefore, the authors suggested that the combination of HMA/VEN was feasible and safe in very old patients but may require dose reduction and cycle extensions for long-term tolerability [135]. Therefore, for older patients with AML, AZA/VEN has portrayed remarkable changes in the therapeutic paradigm, becoming a treatment regimen currently recognized as the established standard of care frontline regimen in this setting [1,25,67,123]. Ongoing research through *BCL2* gene characterization will provide insights into the emergence of its variants and the mechanism of venetoclax resistance at initial treatment of the lack of response at relapse, as well as the basis of the hopeful development of novel treatment approaches in AML R/R patients receiving this agent [71,81,82,136].

### 4.2. Hedgehog Pathway Inhibition

The overactive Hedgehog pathway represents a significant therapeutic target in AML [137,138]. Glasdegib, an inhibitor of the Hedgehog pathway, targets the Smoothened protein, a transmembrane protein that mediates Hedgehog signaling, gained its approval in association with low-dose cytarabine (LDAC) in ND AML patients over 75 years old who are not eligible for ICT due to age or significant health issues [138]. In a controlled study involving 132 AML patients, 88 received the combination treatment of glasdegib and LDAC, while 44 received LDAC alone. Compared to LDAC alone, glasdegib plus LDAC led to a significantly longer OS and a higher CR rate (8.8 vs. 4.9 months and 17% vs. 1%, respectively) [138].

### 4.3. IDH1/2 Inhibition

Other than these therapeutic options relying on dysregulated biological activities, different treatment modalities have been developed based on recognizing somatic mutations druggable by specific targeted agents [16,17,107,139]. With this regard, the approval of *IDH1* inhibitors for patients with *IDH1*-mutated ND AML unsuitable for ICT and those with R/R disease allows a specifically tailored treatment [48,49,50,51,140,141,142,143]. In particular, ivosidenib in combination with azacytidine gained approval for ND IDH1-mutated AML patients aged 75 and above or those with comorbidities following the results of the phase III AGILE trial, which included 146 patients randomly assigned to receive azacytidine plus ivosidenib or azacytidine alone [140]. Compared to the azacytidine monotherapy arm, the study demonstrated a longer PFS and OS (24 vs. 7.9 months) in patients treated with azacytidine and ivosidenib [140]. In addition, the ivosidenib/venetoclax combination, in a clinical trial, was administered as a therapeutic combination alone or in triplet with azacytidine in *IDH1*-mutated MDS, ND, and R/R AML with promising results [141]. Indeed, 63% of treated AML patients achieved MRD negativity. Of note, the 24-month OS duration rates were 50% and 67% in R/R and ND AML, respectively [141]. In addition, an IDH2 inhibitor, such as enasidenib [142], has also been used as monotherapy, allowing for a composite CR of 46%, and in association with azacytidine in patients with the suboptimal response with a further CR rate of 41% [142]. Again, in R/R AML harboring mutant *IDH1*, another *IDH* inhibitor, such as olutasidenib, was approved based on a recently published multicenter clinical trial [143], which evaluated this agent alone or in combination with azacytidine. In this study, olutasidenib allowed for a CR rate of 32%. The median OS for patients with R/R AML was 8.7 months with monotherapy and 12.1 months with combination therapy [143]. Olutasidenib was well tolerated and induced durable responses in older patients with R/R *IDH1*-mutated AML. Therefore, despite the challenges of treating older AML patients who had already failed prior therapy, the results suggest that they can benefit from olutasidenib, providing the rationale for further studies on the therapeutic role of this agent in R/R *IDH1*-mutated AML [143].

### 4.4. FLT3 Inhibition

Another group of therapeutic agents (Table 6) [24,25,26,27,28,29,30] currently having a significant role in targeted therapy for older AML patients is *FLT3* (45–47) inhibitors, particularly concerning the R/R setting [144,145,146,147]. Indeed, mutations in *FLT3* at diagnosis remained at the disease recurrence. Indeed, relapsing patients with *FLT3* negative AML at diagnosis can acquire this mutation, eventually along with other abnormalities, such as *TP53*, *KIT*, *RUNX1*, and *WT1*, at relapse [39,46,47,49]. Therefore, it is of the utmost importance to test for the presence of the *FLT3* mutation in relapsed older AML patients, including those who no longer respond to AZA/VEN combination therapy [146,147]. Indeed, acquiring the *FLT3* mutation during the clonal evolution that characterizes leukemic relapse would make these patients suitable candidates for treatment with an oral, selectively effective *FLT3* inhibitor with single-agent activity, such as gilteritinib, in R/R *FLT3*-mutated AML [39,146,147]. In such a setting, gilteritinib showed a good safety and tolerability profile [39,146,147,148], allowing its use even in fragile or otherwise compromised patients. Therefore, gilteritib represents the most effective salvage option for eligible older patients with R/R AML harboring *FLT3* mutations [39,144,145,146,147]. On the other hand, the efficacy of venetoclax-based salvage therapeutic combination in patients with R/R AML previously treated with *FLT3* or *IDH1/2* inhibitors has been reported [149]. Gilteritinib received approval for patients with R/R *FLT3*-mutated AML based on the results of the ADMIRAL trial [147], which included 371 patients randomized to receive this agent alone (247 patients) or salvage ICT (124 patients). In the gilteritinib arm, the reported OS (9.3 months) was significantly longer than that (5.6 months) recorded in ICT patients [147]. An updated analysis confirmed the survival benefits of gilteritinib compared to ICT, other than its stable safety profile and its role of bridging to allogenic SCT in many responsive patients [148]. Incorporating gilteritinib in therapeutic association with venetoclax [150] and triplet combinations with AZA/VEN [151,152] has been reported with favorable results. The mutual synergies between these agents could be further optimized for clinical benefit in ND AML and R/R AML patients [150,151,152].

### 4.5. More Therapies for Elder AML Patients

The drug tagraxofusp, an anti-CD123 antibody conjugate with toxin for the treatment of BPDN, gained approval for the specific treatment of this rare but severe disease [153,154,155,156,157] based on the results of a single-arm study on 13 treatment-naïve patients, and 54% of them achieved a CR [156]. Since the approval of this agent, research has focused on managing side effects (Table 8), combining therapies to improve outcomes in suitable patients, and developing dosing and combination strategies to reduce toxicities while maintaining effectiveness, especially in older patients. The successful targeting of CD123 in BPDCN has also spurred research into other CD123-positive blood cancers, particularly AML, and promoted the development of new agents targeting CD123 [153,154,155,156,157]. Hence, the approved agents for older AML patients, listed in Table 6, represent significant progress in this field. Clinical trials are exploring their combined use in various therapeutic approaches, such as double or triple combinations, to improve further the management of challenging patients [44,153,154,155,156,157]. Moreover, numerous trials have investigated targeted therapies, including revumenib, a menin inhibitor, in patients with *KMT2A* or *NUP98* rearrangements or *NPM1* mutations [55,56,57,58,59,60,61]. Additionally, specific immunotherapy approaches, such as CAR-T cell therapy [44], and agents like macrolimab [154,158], an anti-CD47 monoclonal antibody, and bispecific antibodies [159], are being studied as potential treatments for AML. Again, some innovative studies are ongoing, exploring other therapeutic targets, such as those linked to already poorly understood metabolic [74,75] and inflammatory [84,85,86,93] aspects of AML. Advances in genomic profiling and molecular characterization have enabled the development of innovative treatment approaches for patients with AML, particularly older and frail individuals [16,17]. Despite limitations and difficulties in the ongoing research, developing these novel and awaited therapies may also offer hope for therapeutic possibilities in challenging situations, such as *TP53*-mutated AML [36,37,160].

### 4.6. Intensive Chemotherapy and Allogeneic SCT

The primary treatment for AML has traditionally been the induction ICT, which involves combining cytarabine and daunorubicin (7 + 3 regimen) [1,27] and still represents the standard of care also in older patients who are able candidates for this therapeutic option based on individual fitness evaluation [119,126,127]. Although the administration of ICT can achieve CR, clinical outcomes are dismal [6], and most older patients with AML are unsuitable for ICT because of advanced age, coexisting health issues, and social concerns [1,25,26,27]. Indeed, ICT for AML may result in lower OS and high mortality rates in older patients, particularly those over 75 years old [6,24,25,26,27,28,29,30,31,32]. However, a recently published study, which included 229 patients > 70 years old with *CBF*-AML treated with ICT and followed long-term in the last two decades, reported an OS of 44% and an EFS of 33%. Based on these encouraging findings, the authors suggested that this subgroup of *CBF*-AML patients could effectively receive ICT approaches [161]. Also, gemtuzumab-ozogamicin, an antibody–drug conjugate targeting CD33 [28,162] to add to traditional ICT, allowed survival benefits that appear limited to patients with favorable-risk *CBF*-AML [28]. In addition, a new treatment option, such as CPX-351, has been introduced to address ICT-related challenges in older patients with AML [163]. This therapeutic combination is a dual-drug liposomal encapsulation exerting its antileukemic action by maintaining a synergistic molar ratio of cytarabine to daunorubicin of 5:1 within the liposome while in circulation [163]. Overall, CPX-351 showed promising benefits among older patients with s-AML or t-AML, resulting in higher response rates and significant improvements in OS and PFS compared to the standard 7 + 3 regimens with a favorable safety profile mainly related to decreased incidence of mucositis and another off-target side effect [1,28,163]. Interestingly, in responsive patients fit for allogeneic SCT, CPX-351 is a feasible bridge to transplant measure [1,28,163]. Notably, low-intensity therapy, such as HMA/VEN and molecularly tailored treatments, has also been proven reliable as a bridge to allogeneic SCT [67,117,145,146,147]. However, due to the frailty of older patients with comorbidities and socioenvironmental factors, the decision to undergo a transplant must be carefully considered by evaluating the treatment-related morbidity associated with allogenic SCT [1,28,117]. Furthermore, treatment decisions based on chronological age alone have been the most common barrier to referring older patients for consideration of allogeneic SCT; concerning this finding, the risk that a significant proportion of older individuals with AML could benefit from allogeneic SCT does not [117]. Therefore, this represents an unmet need, considering that patients undergoing SCT have significantly longer OS than those potentially eligible but not submitted to transplant. However, advancements in lower intensity and less toxic treatments bridging to allogeneic SCT [117], a better understanding of allogenic SCT complications, the increased utilization of unrelated donors, and the development of less intense conditioning strategies have improved transplant outcomes and survival rates over time also for older AML patients [1,117].

### 4.7. The Holistic Approach to Managing AML in Older Patients: Prioritizing Toxicities Management Alongside Quality of Life and Early Palliative

When treating older patients with AML, it is essential to closely monitor and address the adverse effects of new treatments, such as small-molecule inhibitors used alone or in combination therapies (Table 8). For example, the combination therapy AZA/VEN can lead to myelosuppression [25,123,131]. Adjusting the treatment intervals and the duration of venetoclax administration can help reduce the risk. Additionally, dose adjustments of venetoclax should be necessary alongside other medications and antimicrobial prophylaxis, which is advisable at treatment initiation [131]. Granulocyte colony-stimulating factor may help resolve prolonged severe neutropenia since achieving a CR. Therefore, venetoclax dose adjustment is necessary when taken with concomitant cytochrome P3A4 and P-glycoprotein inhibitors. When using IDH1/2 inhibitors, watch out for differentiation syndrome (DS), leukocytosis, and QTc prolongation [48]. Other adverse events include self-limited asymptomatic indirect hyperbilirubinemia with enasidenib, QTc prolongation with ivosidenib, and hepatotoxicity with olutasidenib [48,143]. Midostaurin and gilteritinib are also current therapies for older AML patients, each with its own set of potential adverse effects. The forthcoming targeted agent, such as menin inhibitory, can also cause DS, characterized by symptoms like fever, arthralgias, leukocytosis, pleural or pericardial effusions, and respiratory or renal failure in severe cases [25,55,56,57,58,59,60,61]. Despite advances in AML treatment, patients often experience significant symptom burdens due to the disease itself and the side effects of traditional and novel therapies [164,165,166]. In this regard, palliative care (PC) can alleviate these symptoms and enhance the QoL [164,165]. Additionally, early integrated PC can provide psychological support and counseling to patients and their families to help them cope with the uncertainty and emotional feelings portrayed by the disease [165]. In addition, PC also helps patients navigate complex treatment options, especially when considering the potential benefits of novel agents versus QoL and possible side effects. It is important to note that introducing novel therapies may prolong life, but they do not always lead to a cure [167]. Therefore, incorporating new treatments into patient care plans requires a delicate balance between prolonging life and preserving its quality. In this view, PC care teams should collaborate with hematologists to monitor and address side effects while considering patients’ preferences and goals [165]. Indeed, while these treatments have improved outcomes for many patients, they also present challenges that require a multidisciplinary approach. With this regard, PC focuses on managing symptoms, providing psychosocial support, and planning advanced care to ensure that patients receive comprehensive care aligned with their values and goals as they navigate the complexities of modern AML treatment.

## 5. Summary

The therapeutic scenario of AML has significantly changed over the past 5–10 years due to the approval of several novel treatments. These advancements portrayed a new framework for treating patients with ND and R/R AML, emphasizing the importance of genetic testing and targeted therapies at both stages. Despite these improvements, the mortality rate for AML patients remains high, highlighting the need for better integration of early PC during treatment. With the increasing number of older adults with AML and aging populations, this patient group will ever more represent a significant portion of hematology practices in which ongoing innovative research in AML will bring new therapies and personalized approaches that will continue to improve patient outcomes.

## 6. Key Points

AML patients should receive a quick diagnosis and a proper evaluation of eventually related clinical complications and accompanying comorbidities. Cytogenetic and molecular genetic testing are crucial to diagnose and assess risk accurately. These tests help guide initial treatment, post-remission therapy, and allogeneic SCT and empower hematologists with the necessary information to make informed decisions. In addition, newer and more effective treatments for older AML patients include the AZA/VEN combination, but it can lead to increased myelosuppression, requiring close monitoring and appropriate supportive care.

## 7. Conclusions

AML is a complex and diverse disease, as demonstrated by the expanded genetic and cytogenetic qualifiers in ICC classification [12] and the updated WHO-5 [13] systems. While outcomes have improved through novel therapies [24,25,26,27,28,29,30,31,32] and advances in allogeneic SCT [1,117], the long-term OS remains poor, especially for older patients [1,25]. Therefore, assessing the patient’s fitness [115,119] and staying updated on the latest treatments is essential. Diagnostic tests should detect baseline mutations and monitor changes at relapse. Additionally, MRD assessment can help predict relapse and guide treatment decisions [109,110,111,112]. The introduction of novel targeted drugs has changed our decision-making paradigm, leading to the consideration of efficacy endpoints other than the traditional definition of CR with complete hematological recovery [167]. These changes have included the introduction of specific response categories, such as CR with partial hematological recovery in PB or BM and leukemia-free BM, providing significant benefits for patients who have received non-myelosuppressive therapies. It is important to note that less profound therapeutic responses not integrating a CR state can still be associated with clinically meaningful soothing effects for treating AML with non-myelosuppressive drugs, although less robustly than for CR [167]. Furthermore, novel treatments such as HMAs/VEN can induce long-lasting CR, even persisting after therapy discontinuation [115]. These findings stimulate ongoing investigations exploring the possibility of treatment-free intervals and discontinuation, representing revolutionary concepts in hematology [114,115]. Among the factors determining the best management, it is crucial to consider the patient’s age, expectations, predictable toxicities, impact on patient comorbidities and overall health, and QoL issues [164,165]. Advances in precision medicine [24,25,26,27,28,29,30,31,32] and new treatment options, including novel oral formulation, such as the recently available oral decitabine [168], other than the already many innovative agents administrable by mouth as outpatient regimens [1], make older AML patients ever more manageable, allowing a personalized treatment approach with the valuable aims of reducing the impact of the adverse effects of novel treatments, preserving QoL and ultimately improving outcomes [169].

## Figures and Tables

**Table 1:** Comparison of WHO-5 to ICC 2022 classifications of AMLs [12,13].

Blast Threshold	WHO-5	ICC	Blast Threshold
No cut-off	AMLs with DGA	APL with t (15; 17) (q24.1; q21.2)/*PML: RARA*. APL with others *RARA* rearrangement	10%
	APL with *PML*: *RARA* fusion gene.		
	AML with *RUNX1: RUNX1T1* fusion gene.	AML with t (8/21) (q22; q22.1)/*RUNX1: RUNX1T1* fusion gene.	
	AML with *CBFB: MYH11* fusion gene.	AML with inv (16) (p13.1; q22) or t (16; 16) (p13.1; q22)/*CBFB: MYH11*.	
	AML with KMT2A rearrangements.	AML with t (9; 11) (p21.3; q23.3)/*MLLT3: KTM2A* or other *KMT2A* rearrangements.	
	AML with *DEK: NUP214* fusion gene.	AML with t (6; 9) (p22.3; q34.1)/*DEK: NUP214*.	
	AML with *MECOM* rearrangements	AML with inv (3) (q21.3q; 26.2) or t (3; 3) (q21.3; q26.2)/*GATA: MECOM (EV1)* or other *MECOM* rearrangements	
	AML with other rare translocations (*NUP98*; *RBM15*; *MRTF1*, *DEK*: *NUP214*)		
20%	AML with *BCR*: *ABL1* fusion gene	AML with t (9; 22) (q34.1; q11.2)/*BCR: ABL1*	20%
No cut-off.	AML with NPM1 mutation.		10%
20%	AML with *CEBPA* mutation.	AML with *bZIP CEBPA* in-frame mutation.	20%
	Not classified.	AML with *TP53* mutation.	20%
20%	AML with MDS-related genetic abnormalities.	AML with MDS-related genetic abnormalities (*ASXL1*; *BCOR*, *EZH2*; *RUNX1*; *SF3B1*; *SRSF2*; *STAG2*; *U2AF1*, *ZRSR2*). AML with MDS-related cytogenetic alterations.	20%
20%	AMLs defined by differentiation (Table 2).	AML NOS.	20%
10%	MDS-IB2.	MDS/AML.	10–19%
	Myeloid sarcoma		

WHO-5: Fifth update to the WHO Classification of Haematolymphoid Tumours; ICC: International Consensus Classification of Myeloid Neoplasms and Acute Leukaemias; DGA; defining genetic abnormalities, APL: acute promyelocytic leukemia; *PML*: promyelocytic leukemia gene; *RARA*: retinoic acid receptor alpha gene; AML: acute myeloid leukemia; *RUNX1*: runt-related transcription factor 1; *RUNX1T1*: runt-related transcription factor 1; translocated to, 1 (cyclin D-related); *CBFB*: core binding factor beta; *MYH11*: myosin heavy chain 11; *KMT2A*: (lysine methyltransferase 2A; *MLLT3*: myeloid/lymphoid or mixed-lineage leukemia translocated to chromosome 3 protein; *DEK*: *DEK* proto-oncogene; *NUP214*: nucleoporin 214; *MECOM*: myelodysplasia syndrome 1 (*MDS1*) and ecotropic viral integration site 1 (*EVI1*) complex locus; *GATA2*: *GATA* binding protein 2; *NUP98*: nucleoporin 98, *RBM15*: RNA binding motif protein 15; *MRTF1*; myocardin-related transcription factor 1; *BCR*: breakpoint cluster region protein; *ABL*: Abelson murine leukemia viral oncogene homolog 1: *NPM1*: nucleophosmin 1; *CEBPA*; *CCAAT* enhancer binding protein alpha; *bZIP*: basic leucine zipper region; *TP53*: tumor protein *P53*; MDS: myelodysplastic syndromes; *ASXL1*: additional sex comb-like 1; *BCOR*: Back central optic radius; *EZH2*: enhancer of zeste homolog 2; *SF3B1*: splicing factor 3b subunit 1; *SRSF2*: serine and arginine rich splicing factor 2; *U2AF1*: U2 small nuclear RNA auxiliary factor 1; *ZRSR2*: zinc finger CCCH-type, RNA binding motif and serine/arginine rich 2; NOS; not otherwise specified; MDS: myelodysplastic syndromes; MDS-IB2: MDS with increased blasts.

**Table 2:** WHO-5: AML classification by differentiation features and diagnostic markers [12].

AML Subtype	Diagnostic Criteria
AML with minimal differentiation.	Cytochemistry: MPO and SBB negative blasts (<3%).
	MFC: expression of myeloid antigens (two or more), such as CD13, CD33, and CD117.
AML without maturation.	Morphology: <10% maturing myeloid progenitors of the BM nucleated cells.
	Cytochemistry: ≥3% blasts positive for MPO or SBB and negative for NSE.
	MFC: expression of myeloid antigens (two or more), such as MPO, CD13, CD33, and CD117.
AML with maturation.	Morphology: >10% maturing myeloid progenitors and <20% of the monocytic lineage cells of the BM nucleated cells.
	Cytochemistry: ≥3% blasts positive for MPO or SBB.
	MFC: expression of myeloid antigens (two or more), such as MPO, CD13, CD33, and CD117.
Acute basophilic leukemia.	Morphology: blasts and mature/immature basophils.
	Cytochemistry. Basophils: metachromasia on toluidine blue staining. Blasts: negative for MPO, SBB, and NSA.
	MFC: negative CD117 (to exclude mast cell leukemia).
Acute myelomonocytic leukemia.	Morphology: ≥20% monocytes or their precursors and ≥20% maturing granulocytic cells.
	Cytochemistry and/or MFC: ≥3% of MPO-positive blasts.
Acute monocytic leukemia.	Morphology: ≥80% of monocytes and/or their precursors (monoblasts and/or promonocytes); ≤20% of maturing granulocytic cells.
	MFC/cytochemistry: expression of monocytic antigens (two or more), such as CD11c, CD14, CD36, and CD64, on blasts and promonocytes or their NSE positivity.
Acute erythroid leukemia.	Morphology: erythroid predominance in the BM (>80% of BM cellularity); >30% of immature erythroid (proerythroblasts).
Acute megakaryoblastic leukemia.	MFC: expression of one or more platelet GPs: CD41(GP IIb), CD61 (GP IIIa), or CD42b (GP Ib).

AML: acute myeloid leukemia; MPO: myeloperoxidase; SBB: Sudan Black; MFC: multiparameter flow cytometry; BM: bone marrow; NSE: nonspecific esterase; GP: glycoproteins. Taken and adapted from [12].

**Table 3:** Critical clinical differences between ELN 2022 and ICC compared to WHO-5 classifications of myeloid neoplasms [12,13,16].

	ELN 2022 and ICC 2022	WHO-5
MDS/AML (without AML defining genetic alterations).	10–19% blasts	Designated as MDS-IB2 (10–19% BM or 5–19% PB or Auer Roads).
AML with antecedent MDS, MDS/MPM, or prior exposure to therapy.	MDS was added as a diagnostic qualifier.	Included as a separate entity, “AML-MR”.
AML with *NPM1* mutations, KMT2A rearrangement, *MECOM* rearrangement, and *NUP98* rearrangement.	Requires ≥ 10% blasts in BM or PB.	It can be diagnosed irrespective of blast count.
AML with *CEPA* mutation.	Requires ≥ blasts in BM or PB.	Requires ≥ 20% blasts in BM or PB. Includes bi-allelic and bzip mutations.
*TP53* mutation.	Included separately in the hierarchical classification.	Not included in a separate entity for AML.
Therapy-related.	Added as a diagnostic qualifier.	Included as a separate entity, “AML pCT”.

**Table 4:** ELN 2022 risk classification by genetics at initial diagnosis of AML [16].

Risk category	Favorable	Genetic abnormalities
		t (8; 21) (q22; q22.1)/*RUNX1: RUNX1T1* °*.
		inv (16) (p13.1q22) or t (16; 16) (p13.1; q22)/*CBFB*: *MYH11* °*.
		Mutated *NPM1* °, ^ without *FLT3-ITD*.
		*bZIP* in-frame mutated *CEBPA* °°.
	Intermediate	Mutated *NPM1* °, * with *FLT3-ITD*.
		Wild-type *NPM1* with *FLT3-ITD* (without ARG).
		t (9; 11) (p21.3; q23.3)/*MLLT3: KMT2A* °
		Cytogenetic and/or molecular abnormalities not classified as favorable or adverse.
	Adverse	t (6; 9) (p23.3; q34.1)/*DEK: NUP214*.
		t (v; 11q23.3)/*KMT2A*-rearranged °°°.
		t (9; 22) (q34.1; q11.2)/*BCR: ABL1*.
		t (8; 16) (p11.2; p13.3)/*KAT6A: CREBBP*.
		inv (3) (q21.3q26.2) or t (3; 3) (q21.3; q26.2)/*GATA2*, *MECOM(EVI1)*.
		t (3q26.2; v)/*MECOM(EVI1)*-rearranged.
		Monosomy 5 or del(5q); −7; −17/abn(17p).
		Complex karyotype ^^ monosomic karyotype ^^.
		Mutated *ASXL1*, *BCOR*, *EZH2*, *RUNX1*, *SF3B1*, *SRSF2*, *STAG2*, *U2AF1*, and/or *ZRSR2* ^^^.
		Mutated *TP53* ***.

° Mainly based on results observed in ICT patients. Based on the results from analyses of MRD, initial risk assignment may change during the treatment. * Concurrent *KIT* and/or *FLT3* gene mutation does not alter risk categorization. ^ AML with *NPM1* mutation and adverse-risk cytogenetic abnormalities are categorized as adverse-risk. °° Only in-frame mutations affecting the basic leucine zipper (*bZIP*) region of *CEBPA* have been associated with favorable outcomes. The presence of t (9; 11) (p21.3; q23.3) takes precedence over rare, concurrent adverse risk gene mutations. °°° Excluding *KMT2A* PTD. ^^ Complex karyotype: > or = 3 unrelated chromosome abnormalities without other class-defining recurring genetic abnormalities; excludes hyperdiploid karyotypes with three or more trisomies (or polysomies) without structural abnormalities. These markers should not be used as adverse prognostic markers if they co-occur with favourable-risk AML subtypes. *** *TP53* mutation at a variant allele fraction of at least 10%, irrespective of the *TP53* allelic status (mono- or biallelic mutation); *TP53* mutations are significantly associated with AML with complex and monosomic karyotype. Taken and adapted from [13]. ELN: European Leukaemia Net; AML: acute myeloid leukemia; ARG: adverse-risk gene; ICT: intensive chemotherapy; MRD: measurable residual disease; PTD: partial tandem duplication. ^^^ Adversly prognostic gene mutations.

**Table 5:** ELN 2024 classification by genetic markers for patients receiving less intensive therapy [122].

Risk Category	Genetic Marker	Median OS (Months)
Favorable	Mutated *NPM1* (*FLT3-ITD* ^neg^, *NRAS* ^wt^, *KRAS* ^wt^, *TP53* ^wt^)	39
	Mutated *IDH2* (*FLT3-ITD* ^neg^, *NRAS* ^wt^, *KRAS* ^wt^, *TP53* ^wt^)	37
	Mutated *IDH1* (*TP53* ^wt^)	29
	Mutated *DDX41*	>24
	AML with MDS-related gene mutations (*FLT3*-*ITD* ^neg^, *NRAS* ^wt^, *KRA* ^Swt^, *TP53* ^wt^)	23
Intermediate	AML with MDS-related gene mutations (*FLT3*-*ITD* ^pos^ and/or *NRAS* ^mut^ and/or *KRAS* ^mut^; TP53 ^wt^)	13
	Other cytogenetic and molecular abnormalities (*FLT3-ITD* ^pos^ and/or *NRAS* ^mut^ and/or *KRAS* ^mut^; TP*53* ^wt^)	12
Adverse	Mutated *TP53*	5–8

^neg^: negative; ^wt^: wild type; ^pos^: positive; ^mut^: mutated.

**Table 6:** Current targeted therapies in AML involve older patients [24,25,26,27,28,29,30,31,32].

Therapeutic Mechanisms and Biological Targets		Therapeutic Agent	Indications
Antiapoptotic by inhibition of *BCL2* overexpression		Venetoclax	ND AML in patients > 75 years old or with comorbidities in combination with HMA or LODAC
*FLT3*	*FLT-3 ITD* *FLT-3 TKD*	Midostaurin, Quizartinib	Frontline, in combination with ICT
		Gilteritinib	R/R setting
		Sorafenib	Maintenance following consolidation
*IDH1*	*IDH1*	Ivosidenib	ND AML in patients > 75 years old or with comorbidities; R/R setting
		Olutasidenib	R/R setting
*IDH2*	*IDH2*	Enasidenib	R/R setting
Inhibition of Hedgehog pathway		Glasdegib	Adults older than 75 years who have comorbidities.
ICT with liposomal compounds in s-AML and t-AML		CPX-351	As induction ICT for ND s-AML and t-AML
Anti-CD33 monoclonal antibodies		GO	During induction, ICT for CD33-positive AML or as a single agent in the R/R setting.
Targeting CD123 membrane receptor, cell death via disruption of intracellular protein synthesis by CD123 binding and internalization of the drug		Tagraxofusp (anti-CD123 conjugate with toxin)	Treatment of BPDCN

AML: acute myeloid leukemia; ND: newly diagnosed; HMA; hypomethylating agents; LODAC: low dose of cytarabine; *BCL2*: *B-Cell Lymphoma 2*; *FLT3*; fms related receptor tyrosine kinase 3; *ITD*: internal tandem duplications; *TKD* tyrosine kinase domain mutation; ICT: intensive chemotherapy; R/R: relapse/refractory; *IDH1*: isocitrate dehydrogenase (*IDH*)-1 mutation; *IDH-2* mutation; s-AML: secondary AML; t-AML: therapy-related AML; CPX-351: liposomal encapsulation of cytarabine and daunorubicin at a fixed 5:1 synergistic molar ratio; GO: gentuzumab ozogamicin; BPDCN: blastic plasmocytoid dendritic neoplasms.

**Table 7:** Ongoing recruiting trials involving older AML.

Clinical Study	ClinicalTrials.Gov Identifier
Investigating The Prognostic Significance Of Malnutrition And Sarcopenia in Older Adults with Acute Myeloid Leukemia.	NCT05458258
A Pilot Randomized Controlled Trial of a Patient-Centered Communication Tool (UR-GOAL) for Older Patients With Acute Myeloid Leukemia, Their Caregivers, and Their Oncologists.	NCT05335369
Allogeneic Hematopoietic Cell Transplantation Versus Best Available Standard of Care Therapy in Elderly Patients With Acute Myeloid Leukemia: a Randomized Phase 3 Trial.	NCT04822766
A Randomized Phase II Study of Venetoclax and HMA-Based Therapies for the Treatment of Older and Unfit Adults With Newly Diagnosed FLT3-Mutated Acute Myeloid Leukemia (AML): A myelomatch Treatment Trial.	NCT06317649
Phase I/II Clinical Trial Assessing the Combination of Sulfasalazine With Standard of Care Induction Therapy in Newly Diagnosed Acute Myeloid Leukemias (AML) Patients 60 Years or Older- the SALMA Study.	NCT05580861
A Phase Ib Trial of Azacitidine, Venetoclax and Allogeneic NK Cells for Acute Myeloid Leukemia (ADVENT-AML).	NCT05834244
Relatlimab With Nivolumab and 5-Azacytidine for the Treatment of AML (AARON).	NCT04913922
Dual Growth Factor (rhtpo + G-CSF) and Chemotherapy Combination Regimen for Elderly Patients with Acute Myeloid Leukemia: A Phase II Single-Arm Multicenter Study.	NCT05258799
Dual Growth Factor (rhtpo + G-CSF) and Chemotherapy Combination Regimen in Acute Myeloid Leukemia: Study Protocol for a Randomized Controlled Trial.	NCT05382390
A Prospective, Single-arm, Open-label, Non-interventional, Multi-centre, Post Marketing Surveillance (PMS) Study of Mylotarg^®^.	NCT05189639
Randomized, Sequential, Open-Label Study to Evaluate the Efficacy of IDH Targeted/Non-Targeted Versus Non-targeted/IDH-targeted Approaches in the Treatment of Newly Diagnosed IDH Mutated AML Patients Not Candidates for Intensive Induction Therapy (I-DATA Study).	NCT05401097
Dynamics of Resistance Emergence to Azacitidine-based Therapies in Acute Myeloid Leukemia.	NCT06225128
Phase IA/B Combination Study of ADI-PEG 20, Venetoclax and Azacitidine in Patients with Acute Myeloid Leukemia (AML).	NCT05001828
The Feasibility of Telehealth-Based Palliative Care Intervention and Digital Symptom Monitoring on Patients With AML Receiving Low-Intensity Induction Therapy.	NCT04885127
An Investigator-Sponsored Randomized Phase II Study of Selinexor in Combination With Induction/Consolidation Therapy in Acute Myeloid Leukemia Patients.	NCT02835222
A Prospective Non-interventional Study Documenting the Management and Outcomes of Adult Patients With Acute Myeloid Leukemia (AML).	NCT04777916
Integrative “Omics” Approaches for Leukemia Target Identification and Matched Therapeutic Intervention.	NCT06626893
Maintenance Treatment With Oral Azacitidine for Patients With de Novo AML Including t-AML and AML-MRC in First Remission After CPX-351.	NCT06349239
Phase II Study of Maintenance Ruxolitinib After Allogeneic Stem Cell Transplantation for Older Patients With Acute Myeloid Leukemia (AML) or Myelodysplastic Syndrome (MDS) in Complete Remission.	NCT03286530
Do Decreased Dietary Fat and Increased Fiber Reduce Recurrence of Clostridioides Difficile Infection in Oncology Patients?	NCT04940468
A Telehealth Advance Care Planning Intervention for Older Patients With Myeloid Malignancies: A Pilot Randomized Controlled Trial.	NCT05875805
Phase 1a/1b Study of Itacitinib (INCB039110) for Cytokine Release Syndrome Prevention and Minimization of Immunosuppression Following Nonmyeloablative Related Partially HLA-mismatched Peripheral Blood Stem Cell Transplant (PBSCT) With High-dose Posttransplantation Cyclophosphamide in Older Patients (Age 60 Years).	NCT05823571
Prospective, Observational Study of the Role of Primary Antifungal Prophylaxis to Prevent Invasive Aspergillosis in Elderly Patients With Acute Myeloid Leukemia Undergoing Consolidation Therapy.	NCT06382922
A Master Protocol for Biomarker-Based Treatment of AML (The Beat AML Trial).	NCT03013998
Phase 1 Trial for Patients With Advanced Hematologic Malignancies Undergoing Reduced Intensity Allogeneic HCT With a T-cell Depleted Graft With Infusion of Conventional T-cells and Regulatory T-cells.	NCT05088356
A Phase II Trial of HSCT for the Treatment of Patients With Fanconi Anemia Lacking a Genotypically Identical Donor, Using a Risk-Adjusted Chemotherapy Only Cytoreduction With Busulfan, Cyclophosphamide and Fludarabine.	NCT02143830
A Single Arm Phase II Trial to Assess Cobicistat Boosted Venetoclax in Combination With Azacitidine (sc) in Adult Patients With Newly Diagnosed Acute Myeloid Leukaemia (AML) Who Are Not Considered Candidates for Intensive Treatment Regimens.	NCT06014489
Carolina Senior: UNC Registry for Older Cancer Patients.	NCT01137825
Combined Haploidentical Reduced Intensity Bone Marrow and Kidney Transplantation for Patients With Chronic Kidney Disease and Advanced Hematological Disorders.	NCT01758042
Collection of Blood, Bone Marrow, Skin, Saliva, and Stool Samples From Healthy Volunteers Used for Comparative Analysis of Myeloid Malignancies.	NCT05588154
A Phase I/II Trial of Eltanexor (KPT-8602) With Inqovi (Decitabine-Cedazuridine) in High-Risk Myelodysplastic Syndromes.	NCT05918055
Phase I/II Trial to Determine the Lowest Effective Dose of Post-Transplantation Cyclophosphamide in Combination With Sirolimus and Mycophenolate Mofetil as Graft-Versus-Host Disease Prophylaxis After Reduced Intensity Conditioning and Peripheral Blood Stem Cell Transplantation.	NCT05436418
Source: https://clinicaltrials.gov/ (accessed on 16 October 2024)	

**Table 8:** Main toxicities by approved novel therapies in older AML patients [1,25,28,131].

Therapeutic Agent		Most Common Toxicities and Comments
Venetoclax (ND AML in patients > 75 years old or with comorbidities in combination with HMA or LODAC)		Myelosuppression, notably prolonged neutropenia, could be managed by prolonging treatment intervals and using antimicrobial prophylaxis. G-CSF will be allowed in patients with AML in remission.
*FLT-3 ITD* *FLT-3 TKD*	Midostaurin, Quizartinib (Frontline, in combination with ICT)	GI side effects. The survival benefit of quizartinib was limited to patients younger than 60. There is a high risk of early mortality in older patients.
	Gilteritinib (R/R setting)	Differentiation syndrome, long QT syndrome, and posterior reversible encephalopathy.
*IDH1*	Ivosidenib (ND and R/R AML)	Differentiation syndrome, long QT syndrome.
	Olutasidenib (R/R setting)	Differentiation syndrome, hepatotoxicity.
*IDH2*	Enasidenib (R/R setting)	Differentiation syndrome, hyperbilirubinemia.
Glasdegib (adults older than 75 years who have comorbidities).		Black, tarry stools, bleeding gums, chest pain, chills, confusion, and cough.
CPX-351 (as induction ICT for ND s-AML and t-AML).		Myelosuppression, prolonged neutropenia.
Tagraxofusp (anti-CD123 conjugate with toxin). Treatment of BPDCN.		CLS, nausea, tiredness (fatigue), fever, swelling in your legs or feet, and weight gain.

AML: acute myeloid leukemia; ND: newly diagnosed; HMA: hypomethylating agents; LODAC: low dose of cytarabine; *FLT3*; fms related receptor tyrosine kinase 3; *ITD*: internal tandem duplications; *TKD* tyrosine kinase domain mutation; ICT: intensive chemotherapy; GI: gastrointestinal; R/R: relapse/refractory; *IDH1*: isocitrate dehydrogenase (*IDH*)-1 mutation; *IDH-2* mutation; s-AML: secondary AML; t-AML: therapy-related AML; CPX-351: liposomal encapsulation of cytarabine and daunorubicin at a fixed 5:1 synergistic molar ratio; BPDCN: blastic plasmocytoid dendritic neoplasms. CLS: capillary leak syndrome.

## Data Availability

Reported references from the available English Literature.

## References

[1] Venugopal S., Sekeres M.A. (2024). Contemporary Management of Acute Myeloid Leukemia: A Review. JAMA Oncol..

[2] Wachter F., Pikman Y. (2024). Pathophysiology of Acute Myeloid Leukemia. Acta Haematol..

[3] Shukla M., Abdul-Hay M., Choi J.H. (2024). Molecular Features and Treatment Paradigms of Acute Myeloid Leukemia. Biomedicines.

[4] Bataller A., DiNardo C.D., Bazinet A., Daver N.G., Maiti A., Borthakur G., Short N., Sasaki K., Jabbour E.J., Issa G.C. (2024). Targetable genetic abnormalities in patients with acute myeloblastic leukemia across age groups. Am. J. Hematol..

[5] Aung M.M.K., Mills M.L., Bittencourt-Silvestre J., Keeshan K. (2021). Insights into the molecular profiles of adult and paediatric acute myeloid leukaemia. Mol. Oncol..

[6] Han H.J., Choi K., Suh H.S. (2024). Impact of aging on acute myeloid leukemia epidemiology and survival outcomes: A real-world, population-based longitudinal cohort study. PLoS ONE.

[7] Snaith O., Poveda-Rogers C., Laczko D., Yang G., Morrissette J.J.D. (2024). Cytogenetics and genomics of acute myeloid leukemia. Best Pract. Res. Clin. Haematol..

[8] Guijarro F., Garrote M., Villamor N., Colomer D., Esteve J., López-Guerra M. (2023). Novel Tools for Diagnosis and Monitoring of AML. Curr. Oncol..

[9] Duncavage E.J., Bagg A., Hasserjian R.P., DiNardo C.D., Godley L.A., Iacobucci I., Jaiswal S., Malcovati L., Vannucchi A.M., Patel K.P. (2022). Genomic profiling for clinical decision making in myeloid neoplasms and acute leukemia. Blood.

[10] Mishra S.K., Millman S.E., Zhang L. (2023). Metabolism in acute myeloid leukemia: Mechanistic insights and therapeutic targets. Blood.

[11] Jones C.L., Inguva A., Jordan C.T. (2021). Targeting Energy Metabolism in Cancer Stem Cells: Progress and Challenges in Leukemia and Solid Tumors. Cell Stem Cell.

[12] Khoury J.D., Solary E., Abla O., Akkari Y., Alaggio R., Apperley J.F., Bejar R., Berti E., Busque L., Chan J.K.C. (2022). The 5th edition of the World Health Organization Classification of Haematolymphoid Tumours: Myeloid and Histiocytic/Dendritic Neoplasms. Leukemia.

[13] Arber D.A., Orazi A., Hasserjian R.P., Borowitz M.J., Calvo K.R., Kvasnicka H.M., Wang S.A., Bagg A., Barbui T., Branford S. (2022). International Consensus Classification of Myeloid Neoplasms and Acute Leukemias: Integrating morphologic, clinical, and genomic data. Blood.

[14] Appelbaum F.R. (2023). WHO, what, when, where, and why: New classification systems for acute myeloid leukemia and their impact on clinical practice. Best Pract. Res. Clin. Haematol..

[15] Falini B., Martelli M.P. (2023). Comparison of the International Consensus and 5th WHO edition classifications of adult myelodysplastic syndromes and acute myeloid leukemia. Am. J. Hematol..

[16] Döhner H., Wei A.H., Appelbaum F.R., Craddock C., DiNardo C.D., Dombret H., Ebert B.L., Fenaux P., Godley L.A., Hasserjian R.P. (2022). Diagnosis and management of AML in adults: 2022 recommendations from an international expert panel on behalf of the ELN. Blood.

[17] Pollyea D.A., Altman J.K., Assi R., Bixby D., Fathi A.T., Foran J.M., Gojo I., Hall A.C., Jonas B.A., Kishtagari A. (2023). Acute Myeloid Leukemia, Version 3.2023, NCCN Clinical Practice Guidelines in Oncology. J. Natl. Compr. Cancer Netw..

[18] Catalano G., Zaza A., Banella C., Pelosi E., Castelli G., de Marinis E., Smigliani A., Travaglini S., Ottone T., Divona M. (2023). MCL1 regulates AML cells metabolism via direct interaction with HK2. Metabolic signature at onset predicts overall survival in AMLs’ patients. Leukemia.

[19] Wang Y.H., Orgueira A.M., Lin C.C., Yao C.Y., Lo M.Y., Tsai C.H., de la Fuente Burguera A., Hou H.A., Chou W.C., Tiene H.F. (2024). Stellae-123 gene expression signature improved risk stratification in taiwanese acute myeloid leukemia patients. Sci. Rep..

[20] Lee Y., Baughn L.B., Myers C.L., Sachs Z. (2024). Machine learning analysis of gene expression reveals TP53 Mutant-like AML with wild type TP53 and poor prognosis. Blood Cancer J..

[21] Alhajahjeh A., Nazha A. (2024). Unlocking the Potential of Artificial Intelligence in Acute Myeloid Leukemia and Myelodysplastic Syndromes. Curr. Hematol. Malig. Rep..

[22] Didi I., Alliot J.M., Dumas P.Y., Vergez F., Tavitian S., Largeaud L., Bidet A., Rieu J.B., Luquet I., Lechevalier N. (2024). Artificial intelligence-based prediction models for acute myeloid leukemia using real-life data: A DATAML registry study. Leuk. Res..

[23] Park S., Kim T.Y., Cho B.S., Kwag D., Lee J.M., Kim M., Kim Y., Koo J., Raman A., Kim T.K. (2024). Prognostic value of European LeukemiaNet 2022 criteria and genomic clusters using machine learning in older adults with acute myeloid leukemia. Haematologica.

[24] Wysota M., Konopleva M., Mitchell S. (2024). Novel Therapeutic Targets in Acute Myeloid Leukemia (AML). Curr. Oncol. Rep..

[25] Alsouqi A., Geramita E., Im A. (2023). Treatment of Acute Myeloid Leukemia in Older Adults. Cancers.

[26] Choi J.H., Shukla M., Abdul-Hay M. (2023). Acute Myeloid Leukemia Treatment in the Elderly: A Comprehensive Review of the Present and Future. Acta Haematol..

[27] Roman Diaz J.L., Vazquez Martinez M., Khimani F. (2024). New Approaches for the Treatment of AML beyond the 7+3 Regimen: Current Concepts and New Approaches. Cancers.

[28] Abaza Y., McMahon C., Garcia J.S. (2024). Advancements and Challenges in the Treatment of AML. Am. Soc. Clin. Oncol. Educ. Book.

[29] Bhansali R.S., Pratz K.W., Lai C. (2023). Recent advances in targeted therapies in acute myeloid leukemia. J. Hematol. Oncol..

[30] Zimmer M., Kadia T. (2023). Approach to the Older Patient with Acute Myeloid Leukemia. Curr. Oncol. Rep..

[31] Bhatia K., Sandhu V., Wong M.H., Iyer P., Bhatt S. (2024). Therapeutic biomarkers in acute myeloid leukemia: Functional and genomic approaches. Front. Oncol..

[32] Auerbach S., Puka B., Golla U., Chachoua I. (2024). Recent Advances towards the Understanding of Secondary Acute Myeloid Leukemia Progression. Life.

[33] Hall T., Gurbuxani S., Crispino J.D. (2024). Malignant progression of pre-leukemic disorders. Blood.

[34] Niscola P., Gianfelici V., Giovannini M., Piccioni D., Mazzone C., de Fabritiis P. (2024). Latest Insights and Therapeutic Advances in Myelodysplastic Neoplasms. Cancers.

[35] Fabre M.A., Vassiliou G.S. (2024). The lifelong natural history of clonal hematopoiesis and its links to myeloid neoplasia. Blood.

[36] Molica M., Mazzone C., Niscola P., de Fabritiis P. (2021). TP53 Mutations in Acute Myeloid Leukemia: Still a Daunting Challenge?. Front. Oncol..

[37] Zhao Y., Chen W., Yu J., Pei S., Zhang Q., Shi J., Huang H., Zhao Y. (2024). TP53 in MDS and AML: Biological and clinical advances. Cancer Lett..

[38] Santini V., Stahl M., Sallman D.A. (2024). TP53 Mutations in Acute Leukemias and Myelodysplastic Syndromes: Insights and Treatment Updates. Am. Soc. Clin. Oncol. Educ. Book.

[39] Kim N., Hahn S., Choi Y.J., Cho H., Chung H., Jang J.E., Lyu C.J., Lee S.T., Choi J.R., Cheong J.W. (2024). Comprehensive insights into AML relapse: Genetic mutations, clonal evolution, and clinical outcomes. Cancer Cell Int..

[40] Luque Paz D., Jouanneau-Courville R., Riou J., Ianotto J.C., Boyer F., Chauveau A., Renard M., Chomel J.C., Cayssials E., Gallego-Hernanz M.P. (2020). Leukemic evolution of polycythemia vera and essential thrombocythemia: Genomic profiles predict time to transformation. Blood Adv..

[41] Zhang A., Liu W., Guo X., Jia H., Wei Y., Can C., He N., Ji C., Ma D. (2024). Genetic variations in DNA excision repair pathway contribute to the chemosensitivity and prognosis of acute myeloid leukemia. Clin. Chim. Acta.

[42] Angelini D.F., Ottone T., Guerrera G., Lavorgna S., Cittadini M., Buccisano F., De Bardi M., Gargano F., Maurillo L., Divona M. (2015). A Leukemia-Associated CD34/CD123/CD25/CD99+ Immunophenotype Identifies FLT3-Mutated Clones in Acute Myeloid Leukemia. Clin. Cancer Res..

[43] Travaglini S., Ottone T., Angelini D.F., Fiori V., Dominici S., Noguera N.I., Śniegocka M., Antonelli S., Irno Consalvo M.A., De Bardi M. (2022). CD99 as a novel therapeutic target on leukemic progenitor cells in FLT3-ITD^mut^ AML. Leukemia.

[44] Gao C., Li X., Xu Y., Zhang T., Zhu H., Yao D. (2024). Recent advances in CAR-T cell therapy for acute myeloid leukemia. J. Cell Mol. Med..

[45] Macečková D., Vaňková L., Holubová M., Jindra P., Klieber R., Jandová E., Pitule P. (2024). Current knowledge about FLT3 gene mutations, exploring the isoforms, and protein importance in AML. Mol. Biol. Rep..

[46] Kennedy V.E., Smith C.C. (2023). FLT3 targeting in the modern era: From clonal selection to combination therapies. Int. J. Hematol..

[47] Grob T., Sanders M.A., Vonk C.M., Kavelaars F.G., Rijken M., Hanekamp D.W., Gradowska P.L., Cloos J., Fløisand Y., van Marwijk Kooy M. (2023). Prognostic Value of FLT3-Internal Tandem Duplication Residual Disease in Acute Myeloid Leukemia. J. Clin. Oncol..

[48] Fruchtman H., Avigan Z.M., Waksal J.A., Brennan N., Mascarenhas J.O. (2024). Management of isocitrate dehydrogenase 1/2 mutated acute myeloid leukemia. Leukemia.

[49] Lachowiez C.A., DiNardo C.D., Loghavi S. (2023). Molecularly Targeted Therapy in Acute Myeloid Leukemia: Current Treatment Landscape and Mechanisms of Response and Resistance. Cancers.

[50] Figueroa M.E., Abdel-Wahab O., Lu C., Ward P.S., Patel J., Shih A., Li Y., Bhagwat N., Vasanthakumar A., Fernandez H.F. (2010). Leukemic IDH1 and IDH2 mutations result in a hypermethylation phenotype, disrupt TET2 function, and impair hematopoietic differentiation. Cancer Cell.

[51] Bewersdorf J.P., Shimony S., Shallis R.M., Liu Y., Berton G., Schaefer E.J., Zeidan A.M., Goldberg A., Stein E., Marcucci G. (2024). Combination therapy with hypomethylating agents and venetoclax versus intensive induction chemotherapy in IDH1- or IDH2-mutant newly diagnosed acute myeloid leukemia-A multicenter cohort study. Am. J. Hematol..

[52] Issa G.C., Zarka J., Sasaki K., Qiao W., Pak D., Ning J., Short N.J., Haddad F., Tang Z., Patel K.P. (2021). Predictors of outcomes in adults with acute myeloid leukemia and KMT2A rearrangements. Blood Cancer J..

[53] Falini B., Martelli M.P., Brunetti L., Gjertsen B.T., Andresen V. (2023). The NPM1 mutant defines AML irrespective of blast count. Am. J. Hematol..

[54] Falini B., Martelli M.P., Brunetti L. (2023). Mutant NPM1: Nuclear export and the mechanism of leukemogenesis. Am. J. Hematol..

[55] Thomas X. (2024). Small Molecule Menin Inhibitors: Novel Therapeutic Agents Targeting Acute Myeloid Leukemia with KMT2A Rearrangement or NPM1 Mutation. Oncol. Ther..

[56] Candoni A., Coppola G. (2024). A 2024 Update on Menin Inhibitors. A New Class of Target Agents against KMT2A-Rearranged and NPM1-Mutated Acute Myeloid Leukemia. Hematol. Rep..

[57] Kühn M.W.M., Ganser A. (2024). The Menin story in acute myeloid leukaemia-The road to success. Br. J. Haematol..

[58] Issa G.C., Aldoss I., DiPersio J., Cuglievan B., Stone R., Arellano M., Thirman M.J., Patel M.R., Dickens D.S., Shenoy S. (2023). The menin inhibitor revumenib in KMT2A-rearranged or NPM1-mutant leukaemia. Nature.

[59] Rasouli M., Blair H., Troester S., Szoltysek K., Cameron R., Ashtiani M., Krippner-Heidenreich A., Grebien F., McGeehan G., Zwaan C.M. (2023). The MLL-Menin Interaction is a Therapeutic Vulnerability in NUP98-rearranged AML. Hemasphere.

[60] Heikamp E.B., Henrich J.A., Perner F., Wong E.M., Hatton C., Wen Y., Barwe S.P., Gopalakrishnapillai A., Xu H., Uckelmann H.J. (2022). The menin-MLL1 interaction is a molecular dependency in NUP98-rearranged AML. Blood.

[61] Perner F., Stein E.M., Wenge D.V., Singh S., Kim J., Apazidis A., Rahnamoun H., Anand D., Marinaccio C., Hatton C. (2023). MEN1 mutations mediate clinical resistance to menin inhibition. Nature.

[62] Othman J., Meggendorfer M., Tiacci E., Thiede C., Schlenk R., Dillon R., Stasik S., Venanzi A., Bertoli S., Delabesse E. (2023). Overlapping features of therapy-related and de novo NPM1-mutated AML. Blood.

[63] Kühn M.W., Song E., Feng Z., Sinha A., Chen C.W., Deshpande A.J., Cusan M., Farnoud N., Mupo A., Grove C. (2016). Targeting Chromatin Regulators Inhibits Leukemogenic Gene Expression in NPM1 Mutant Leukemia. Cancer Discov..

[64] Lambert M., Jambon S., Bouhlel M.A., Depauw S., Vrevin J., Blanck S., Marot G., Figeac M., Preudhomme C., Quesnel B. (2024). Induction of AML cell differentiation using HOXA9/DNA binding inhibitors as a potential therapeutic option for HOXA9-dependent AML. Hemasphere.

[65] Rodríguez-Medina C., Stuckey R., Bilbao-Sieyro C., Gómez-Casares M.T. (2024). Biomarkers of Response to Venetoclax Therapy in Acute Myeloid Leukemia. Int. J. Mol. Sci..

[66] Mestrum S.G.C., Roanalis B.Y.V., de Wit N.C.J., Drent R.J.M., Boonen B.T., van Hemert W.L.W., Hopman A.H.N., Ramaekers F.C.S., Leers M.P.G. (2024). MDS and AML show elevated fractions of CD34-positive blast cell populations with a high anti-apoptotic versus proliferation ratio. Leuk. Res..

[67] DiNardo C.D., Jonas B.A., Pullarkat V., Thirman M.J., Garcia J.S., Wei A.H., Konopleva M., Döhner H., Letai A., Fenaux P. (2020). Azacitidine and Venetoclax in Previously Untreated Acute Myeloid Leukemia. N. Engl. J. Med..

[68] DiNardo C.D. (2024). Toward an improved understanding of hypomethylating agent and venetoclax therapies. Am. J. Hematol..

[69] Galluzzi L., Kepp O., Tajeddine N., Kroemer G. (2008). Disruption of the hexokinase-VDAC complex for tumor therapy. Oncogene.

[70] Stevens B.M., Jones C.L., Pollyea D.A., Culp-Hill R., D′Alessandro A., Winters A., Krug A., Abbott D., Goosman M., Pei S. (2020). Fatty acid metabolism underlies venetoclax resistance in acute myeloid leukemia stem cells. Nat. Cancer.

[71] Pei S., Pollyea D.A., Gustafson A., Stevens B.M., Minhajuddin M., Fu R., Riemondy K.A., Gillen A.E., Sheridan R.M., Kim J. (2020). Monocytic Subclones Confer Resistance to Venetoclax-Based Therapy in Patients with Acute Myeloid Leukemia. Cancer Discov..

[72] Paudel B.B., Tan S.F., Fox T.E., Ung J., Golla U., Shaw J.J.P., Dunton W., Lee I., Fares W.A., Patel S. (2024). Acute myeloid leukemia stratifies as 2 clinically relevant sphingolipidomic subtypes. Blood Adv..

[73] Pino J.C., Posso C., Joshi S.K., Nestor M., Moon J., Hansen J.R., Hutchinson-Bunch C., Gritsenko M.A., Weitz K.K., Watanabe-Smith K. (2024). Mapping the proteogenomic landscape enables prediction of drug response in acute myeloid leukemia. Cell Rep. Med..

[74] Shi X., Feng M., Nakada D. (2024). Metabolic dependencies of acute myeloid leukemia stem cells. Int. J. Hematol..

[75] Pereira-Vieira J., Weber D.D., Silva S., Barbosa-Matos C., Granja S., Reis R.M., Queirós O., Ko Y.H., Kofler B., Casal M. (2024). Glucose Metabolism as a Potential Therapeutic Target in Cytarabine-Resistant Acute Myeloid Leukemia. Pharmaceutics.

[76] Jones C.L., Stevens B.M., D′Alessandro A., Reisz J.A., Culp-Hill R., Nemkov T., Pei S., Khan N., Adane B., Ye H. (2019). Inhibition of Amino Acid Metabolism Selectively Targets Human Leukemia Stem Cells. Cancer Cell.

[77] Banella C., Catalano G., Travaglini S., Pelosi E., Ottone T., Zaza A., Guerrera G., Angelini D.F., Niscola P., Divona M. (2022). Ascorbate Plus Buformin in AML: A Metabolic Targeted Treatment. Cancers.

[78] Panuzzo C., Jovanovski A., Pergolizzi B., Pironi L., Stanga S., Fava C., Cilloni D. (2020). Mitochondria: A Galaxy in the Hematopoietic and Leukemic Stem Cell Universe. Int. J. Mol. Sci..

[79] Vannini N., Girotra M., Naveiras O., Nikitin G., Campos V., Giger S., Roch A., Auwerx J., Lutolf M.P. (2016). Specification of haematopoietic stem cell fate via modulation of mitochondrial activity. Nat. Commun..

[80] Mattes K., Vellenga E., Schepers H. (2019). Differential redox-regulation and mitochondrial dynamics in normal and leukemic hematopoietic stem cells: A potential window for leukemia therapy. Crit. Rev. Oncol. Hematol..

[81] Hata A.N., Engelman J.A., Faber A.C. (2015). The BCL2 Family: Key Mediators of the Apoptotic Response to Targeted Anticancer Therapeutics. Cancer Discov..

[82] Kaloni D., Diepstraten S.T., Strasser A., Kelly G.L. (2023). BCL-2 protein family: Attractive targets for cancer therapy. Apoptosis.

[83] Deng H., Han Y., Liu L., Zhang H., Liu D., Wen J., Huang M., Zhao L. (2024). Targeting Myeloid Leukemia-1 in Cancer Therapy: Advances and Directions. J. Med. Chem..

[84] Bakhtiyari M., Liaghat M., Aziziyan F., Shapourian H., Yahyazadeh S., Alipour M., Shahveh S., Maleki-Sheikhabadi F., Halimi H., Forghaniesfidvajani R. (2023). The role of bone marrow microenvironment (BMM) cells in acute myeloid leukemia (AML) progression: Immune checkpoints, metabolic checkpoints, and signaling pathways. Cell Commun. Signal..

[85] Minciacchi V.R., Karantanou C., Bravo J., Pereira R.S., Zanetti C., Krack T., Kumar R., Bankov K., Hartmann S., Huntly B.J. (2024). Differential inflammatory conditioning of the bone marrow by acute myeloid leukemia and its impact on progression. Blood Adv..

[86] Filipek-Gorzała J., Kwiecińska P., Szade A., Szade K. (2024). The dark side of stemness—The role of hematopoietic stem cells in development of blood malignancies. Front. Oncol..

[87] Haouas H. (2014). Angiogenesis and acute myeloid leukemia. Hematology.

[88] Zhang H., Sun C., Sun Q., Li Y., Zhou C., Sun C. (2023). Susceptibility of acute myeloid leukemia cells to ferroptosis and evasion strategies. Front. Mol. Biosci..

[89] Bian Y., Li W., Kremer D.M., Sajjakulnukit P., Li S., Crespo J., Nwosu Z.C., Zhang L., Czerwonka A., Pawłowska A. (2020). Cancer SLC43A2 alters T cell methionine metabolism and histone methylation. Nature.

[90] Fan C., Yang X., Yan L., Shi Z. (2024). Oxidative stress is two-sided in the treatment of acute myeloid leukemia. Cancer Med..

[91] Leone R.D., Powell J.D. (2021). Fueling the Revolution: Targeting Metabolism to Enhance Immunotherapy. Cancer Immunol. Res..

[92] Uy G.L., DeAngelo D.J., Lozier J.N., Fisher D.M., Jonas B.A., Magnani J.L., Becker P.S., Lazarus H.M., Winkler I.G. (2024). Targeting hematologic malignancies by inhibiting E-selectin: A sweet spot for AML therapy. Blood Rev..

[93] Vegivinti C.T.R., Keesari P.R., Veeraballi S., Martins Maia C.M.P., Mehta A.K., Lavu R.R., Thakur R.K., Tella S.H., Patel R., Kakumani V.K. (2023). Role of innate immunological/inflammatory pathways in myelodysplastic syndromes and AML: A narrative review. Exp. Hematol. Oncol..

[94] Li Z., Cui J. (2023). Targeting the lactic acid metabolic pathway for antitumor therapy. Mol. Ther. Oncolytics.

[95] Hu Z., Yang Y., Li J., Hu Z. (2024). Genetic mutations and immune microenvironment: Unveiling the connection to AML prognosis. Hematology.

[96] Chen Y., Qiu X., Liu R. (2024). Comprehensive characterization of immunogenic cell death in acute myeloid leukemia revealing the association with prognosis and tumor immune microenvironment. BMC Med. Genom..

[97] Lasry A., Nadorp B., Fornerod M., Nicolet D., Wu H., Walker C.J., Sun Z., Witkowski M.T., Tikhonova A.N., Guillamot-Ruano M. (2023). An inflammatory state remodels the immune microenvironment and improves risk stratification in acute myeloid leukemia. Nat. Cancer.

[98] Cheng F.M., Lo S.C., Lin C.C., Lo W.J., Chien S.Y., Sun T.H., Hsu K.C. (2024). Deep learning assists in acute leukemia detection and cell classification via flow cytometry using the acute leukemia orientation tube. Sci. Rep..

[99] Makishima H., Yoshizato T., Yoshida K., Sekeres M.A., Radivoyevitch T., Suzuki H., Przychodzen B., Nagata Y., Meggendorfer M., Sanada M. (2017). Dynamics of clonal evolution in myelodysplastic syndromes. Nat. Genet..

[100] Warnat-Herresthal S., Perrakis K., Taschler B., Becker M., Baßler K., Beyer M., Günther P., Schulte-Schrepping J., Seep L., Klee K. (2020). Scalable Prediction of Acute Myeloid Leukemia Using High-Dimensional Machine Learning and Blood Transcriptomics. iScience.

[101] Cheng Y., Yang X., Wang Y., Li Q., Chen W., Dai R., Zhang C. (2024). Multiple machine-learning tools identifying prognostic biomarkers for acute Myeloid Leukemia. BMC Med. Inform. Decis. Mak..

[102] Van Galen P., Hovestadt V., Wadsworth M.H., Hughes T.K., Griffin G.K., Battaglia S., Verga J.A., Stephansky J., Pastika T.J., Lombardi Story J. (2019). Single-Cell RNA-Seq Reveals AML Hierarchies Relevant to Disease Progression and Immunity. Cell.

[103] Bruno S., Borsi E., Patuelli A., Bandini L., Mancini M., Forte D., Nanni J., Barone M., Grassi A., Cristiano G. (2024). Tracking Response and Resistance in Acute Myeloid Leukemia through Single-Cell DNA Sequencing Helps Uncover New Therapeutic Targets. Int. J. Mol. Sci..

[104] Lucas F., Hergott C.B. (2023). Advances in Acute Myeloid Leukemia Classification, Prognostication and Monitoring by Flow Cytometry. Clin. Lab. Med..

[105] Arber D.A., Orazi A., Hasserjian R., Thiele J., Borowitz M.J., Le Beau M.M., Bloomfield C.D., Cazzola M., Vardiman J.W. (2016). The 2016 revision to the World Health Organization classification of myeloid neoplasms and acute leukemia. Blood.

[106] Huber S., Baer C., Hutter S., Dicker F., Meggendorfer M., Pohlkamp C., Kern W., Haferlach T., Haferlach C., Hoermann G. (2023). AML classification in the year 2023: How to avoid a Babylonian confusion of languages. Leukemia.

[107] Turkalj S., Radtke F.A., Vyas P. (2023). An Overview of Targeted Therapies in Acute Myeloid Leukemia. Hemasphere.

[108] Zhou Q., Zhao D., Zarif M., Davidson M.B., Minden M.D., Tierens A., Yeung Y.W.T., Wei C., Chang H. (2024). A real-world analysis of clinical outcomes in AML with myelodysplasia-related changes: A comparison of ICC and WHO-HAEM5 criteria. Blood Adv..

[109] Li X., Tong X. (2023). Role of Measurable Residual Disease in Older Adult Acute Myeloid Leukemia. Clin. Interv. Aging.

[110] Tiong I.S., Loo S. (2023). Targeting Measurable Residual Disease (MRD) in Acute Myeloid Leukemia (AML): Moving beyond Prognostication. Int. J. Mol. Sci..

[111] Sun Y., Zhu G., Zhong H. (2024). Minimal residual disease monitoring in acute myeloid leukemia: Focus on MFC-MRD and treatment guidance for elderly patients. Eur. J. Haematol..

[112] Pratz K.W., Jonas B.A., Pullarkat V., Recher C., Schuh A.C., Thirman M.J., Garcia J.S., DiNardo C.D., Vorobyev V., Fracchiolla N.S. (2022). Measurable Residual Disease Response and Prognosis in Treatment-Naïve Acute Myeloid Leukemia With Venetoclax and Azacitidine. J. Clin. Oncol..

[113] Niscola P., Gianfelici V., Giovannini M., Piccioni D., Mazzone C., Fabritiis P. (2024). Very long-term efficacy of venetoclax combined with hypomethylating agents in two AML elderly: Is it the time for treatment discontinuation strategies?. Ann. Hematol..

[114] Garciaz S., Dumas P.Y., Bertoli S., Sallman D.A., Decroocq J., Belhabri A., Orvain C., Aspas Requena G., Simand C., Laribi K. (2024). Outcomes of acute myeloid leukemia patients who responded to venetoclax and azacitidine and stopped treatment. Am. J. Hematol..

[115] Boscaro E., Urbino I., Catania F.M., Arrigo G., Secreto C., Olivi M., D′Ardia S., Frairia C., Giai V., Freilone R. (2023). Modern Risk Stratification of Acute Myeloid Leukemia in 2023: Integrating Established and Emerging Prognostic Factors. Cancers.

[116] Song G.Y., Kim H.J., Kim T., Ahn S.Y., Jung S.H., Kim M., Yang D.H., Lee J.J., Kim M.Y., Cheong J.W. (2024). Validation of the 2022 European LeukemiaNet risk stratification for acute myeloid leukemia. Sci. Rep..

[117] Bazinet A., Kantarjian H., Arani N., Popat U., Bataller A., Sasaki K., DiNardo C.D., Daver N., Yilmaz M., Abbas H.A. (2023). Evolving trends and outcomes in older patients with acute myeloid leukemia including allogeneic stem cell transplantation. Am. J. Hematol..

[118] Jen W.Y., Kantarjian H., Kadia T.M., DiNardo C.D., Issa G.C., Short N.J., Yilmaz M., Borthakur G., Ravandi F., Daver N.G. (2024). Combination therapy with novel agents for acute myeloid leukaemia: Insights into treatment of a heterogenous disease. Br. J. Haematol..

[119] Rossi G., Borlenghi E., Zappasodi P., Lussana F., Bernardi M., Basilico C., Molteni A., Lotesoriere I., Turrini M., Frigeni M. (2024). Adapting the Fitness Criteria for Non-Intensive Treatments in Older Patients with Acute Myeloid Leukemia to the Use of Venetoclax-Hypomethylating Agents Combination-Practical Considerations from the Real-Life Experience of the Hematologists of the Rete Ematologica Lombarda. Cancers.

[120] Hoff F.W., Blum W., Huang Y., Welkie R.L., Swords R., Traer E., Stein E.M., Lin T.L., Archer K.J., Patel P.A. (2024). Beat-AML 2024 ELN-Refined Risk Stratification for Older Adults with Newly Diagnosed AML Given Lower-Intensity Therapy. Blood Adv..

[121] Bataller A., Bazinet A., DiNardo C.D., Maiti A., Borthakur G., Daver N.G., Short N.J., Jabbour E.J., Issa G.C., Pemmaraju N. (2024). Prognostic risk signature in patients with acute myeloid leukemia treated with hypomethylating agents and venetoclax. Blood Adv..

[122] Döhner H., DiNardo C.D., Wei A.H., Löwenberg B., Appelbaum F., Craddock C., Dombret H., Ebert B.L., Fenaux P., Godley L.A. (2024). Genetic risk classification for adults with AML receiving less-intensive therapies: The 2024 ELN recommendations. Blood.

[123] Wei A.H., Loo S., Daver N.G. (2024). How I Treat patients with AML using azacitidine and venetoclax. Blood.

[124] Getz T.M., Bewersdorf J.P., Kewan T., Stempel J.M., Bidikian A., Shallis R.M., Stahl M., Zeidan A.M. (2024). Beyond HMAs: Novel Targets and Therapeutic Approaches. Seminars in Hematology.

[125] Heuser M., Fernandez C., Hauch O., Klibanov O.M., Chaudhary T., Rives V. (2023). Therapies for acute myeloid leukemia in patients ineligible for standard induction chemotherapy: A systematic review. Future Oncol..

[126] Barosi G., Venditti A., Angelucci E., Gobbi M., Pane F., Tosi P., Zinzani P., Tura S. (2013). Consensus-based definition of unfitness to intensive and non-intensive chemotherapy in acute myeloid leukemia: A project of SIE, SIES and GITMO group on a new tool for therapy decision making. Leukemia.

[127] Apolito V., Arrigo G., Vasseur L., Olivi M., Perrone S., Giai V., Secreto C., Di Biase F., De Simone M.C., Copia C. (2023). Validation of SIE/SIES/GITMO consensus criteria for unfitness to predict early mortality and survival in acute myeloid leukemia patients treated with hypomethylating agents and venetoclax. Br. J. Haematol..

[128] Pratz K.W., Jonas B.A., Pullarkat V., Thirman M.J., Garcia J.S., Döhner H., Récher C., Fiedler W., Yamamoto K., Wang J. (2024). Long-term follow-up of VIALE-A: Venetoclax and azacitidine in chemotherapy-ineligible untreated acute myeloid leukemia. Am. J. Hematol..

[129] He H., Wen X., Zheng H. (2024). Efficacy and safety of venetoclax-based combination therapy for previously untreated acute myeloid leukemia: A meta-analysis. Hematology.

[130] Lai C., Bhansali R.S., Kuo E.J., Mannis G., Lin R.J. (2023). Older Adults with Newly Diagnosed AML: Hot Topics for the Practicing Clinician. Am. Soc. Clin. Oncol. Educ. Book.

[131] Wang E.S., Baron J. (2020). Management of toxicities associated with targeted therapies for acute myeloid leukemia: When to push through and when to stop. Hematol. Am. Soc. Hematol. Educ. Program.

[132] Bewersdorf J.P., Shimony S., Shallis R.M., Liu Y., Berton G., Schaefer E.J., Zeidan A.M., Goldberg A.D., Stein E.M., Marcucci G. (2024). Intensive Induction Chemotherapy versus Hypomethylating Agents in Combination with Venetoclax in NPM1-mutant AML. Blood Adv..

[133] Sartor C., Brunetti L., Audisio E., Cignetti A., Zannoni L., Cristiano G., Nanni J., Ciruolo R., Zingarelli F., Ottaviani E. (2023). A venetoclax and azacitidine bridge-to-transplant strategy for NPM1-mutated acute myeloid leukaemia in molecular failure. Br. J. Haematol..

[134] Niscola P., Mazzone C., Fratoni S., Ardu N.R., Cesini L., Giovannini M., Ottone T., Anemona L., Voso M.T., de Fabritiis P. (2023). Acute Myeloid Leukemia with NPM1 Mutation and Disseminated Leukemia Cutis: Achievement of Molecular Complete Remission by Venetoclax/Azacitidine Combination in a Very Old Patient. Acta Haematol..

[135] Madarang E., Lykon J., Zhao W., Sekeres M.A., Bradley T., Chandhok N.S., Taylor J., Venugopal S., Koru-Sengul T., Iyer S.G. (2024). Venetoclax and hypomethylating agents in octogenarians and nonagenarians with acute myeloid leukemia. Blood Neoplasia.

[136] Brown F.C., Wang X., Birkinshaw R.W., Chua C.C., Morley T.D., Kasapgil S., Pomilio G., Blombery P., Huang D.C.S., Czabotar P.E. (2024). Acquired BCL2 variants associated with venetoclax resistance in acute myeloid leukemia. Blood Adv..

[137] Lemos T., Merchant A. (2022). The hedgehog pathway in hematopoiesis and hematological malignancy. Front. Oncol..

[138] Cortes J.E., Heidel F.H., Hellmann A., Fiedler W., Smith B.D., Robak T., Montesinos P., Pollyea D.A., DesJardins P., Ottmann O. (2019). Randomized comparison of low dose cytarabine with or without glasdegib in patients with newly diagnosed acute myeloid leukemia or high-risk myelodysplastic syndrome. Leukemia.

[139] Premnath N., Madanat Y.F. (2023). Paradigm Shift in the Management of Acute Myeloid Leukemia—Approved Options in 2023. Cancers.

[140] Montesinos P., Recher C., Vives S., Zarzycka E., Wang J., Bertani G., Heuser M., Calado R.T., Schuh A.C., Yeh S.P. (2022). Ivosidenib and Azacitidine in IDH1-Mutated Acute Myeloid Leukemia. N. Engl. J. Med..

[141] Lachowiez C.A., Loghavi S., Zeng Z., Tanaka T., Kim Y.J., Uryu H., Turkalj S., Jakobsen N.A., Luskin M.R., Duose D.Y. (2023). A Phase Ib/II Study of Ivosidenib with Venetoclax ± Azacitidine in IDH1-Mutated Myeloid Malignancies. Blood Cancer Discov..

[142] Cai S.F., Huang Y., Lance J.R., Mao H.C., Dunbar A.J., McNulty S.N., Druley T., Li Y., Baer M.R., Stock W. (2024). A study to assess the efficacy of enasidenib and risk-adapted addition of azacitidine in newly diagnosed IDH2-mutant AML. Blood Adv..

[143] Watts J.M., Baer M.R., Yang J., Prebet T., Lee S., Schiller G.J., Dinner S.N., Pigneux A., Montesinos P., Wang E.S. (2023). Olutasidenib alone or with azacitidine in IDH1-mutated acute myeloid leukaemia and myelodysplastic syndrome: Phase 1 results of a phase 1/2 trial. Lancet Haematol..

[144] Bocchia M., Carella A.M., Mulè A., Rizzo L., Turrini M., Abbenante M.C., Cairoli R., Calafiore V., Defina M., Gardellini A. (2022). Therapeutic Management of Patients with FLT3 + Acute Myeloid Leukemia: Case Reports and Focus on Gilteritinib Monotherapy. Pharmacogenomics Pers. Med..

[145] Saburi M., Sakata M., Maruyama R., Kodama Y., Takata H., Miyazaki Y., Kawano K., Wada J., Urabe S., Ohtsuka E. (2023). Gilteritinib as Bridging and Posttransplant Maintenance for Relapsed Acute Myeloid Leukemia with FLT3-ITD Mutation Accompanied by Extramedullary Disease in Elderly. Case Rep. Hematol..

[146] Zhang L.S., Wang J., Xu M.Z., Wu T.M., Huang S.M., Cao H.Y., Sun A.N., Liu S.B., Xue S.L. (2022). Rapid and Efficient Response to Gilteritinib and Venetoclax-Based Therapy in Two AML Patients with FLT3-ITD Mutation Unresponsive to Venetoclax Plus Azacitidine. Onco Targets Ther..

[147] Perl A.E., Martinelli G., Cortes J.E., Neubauer A., Berman E., Paolini S., Montesinos P., Baer M.R., Larson R.A., Ustun C. (2019). Gilteritinib or Chemotherapy for Relapsed or Refractory FLT3-Mutated AML. N. Engl. J. Med..

[148] Perl A.E., Larson R.A., Podoltsev N.A., Strickland S., Wang E.S., Atallah E., Schiller G.J., Martinelli G., Neubauer A., Sierra J. (2022). Follow-up of patients with R/R FLT3-mutation-positive AML treated with gilteritinib in the phase 3 ADMIRAL trial. Blood.

[149] Bewersdorf J.P., Shallis R.M., Derkach A., Goldberg A.D., Stein A., Stein E.M., Marcucci G., Zeidan A.M., Shimony S., DeAngelo D.J. (2023). Venetoclax-based salvage therapy in patients with relapsed/refractory acute myeloid leukemia previously treated with FLT3 or IDH1/2 inhibitors. Leuk. Lymphoma.

[150] Daver N., Perl A.E., Maly J., Levis M., Ritchie E., Litzow M., McCloskey J., Smith C.C., Schiller G., Bradley T. (2022). Venetoclax Plus Gilteritinib for FLT3-Mutated Relapsed/Refractory Acute Myeloid Leukemia. J. Clin. Oncol..

[151] Venugopal S., Watts J. (2023). The future paradigm of HMA + VEN or targeted inhibitor approaches: Sequencing or triplet combinations in AML therapy. Hematol. Am. Soc. Hematol. Educ. Program.

[152] Short N.J., Daver N., Dinardo C.D., Kadia T., Nasr L.F., Macaron W., Yilmaz M., Borthakur G., Montalban-Bravo G., Garcia-Manero G. (2024). Azacitidine, Venetoclax, and Gilteritinib in Newly Diagnosed and Relapsed or Refractory FLT3-Mutated AML. J. Clin. Oncol..

[153] Bordeleau M.E., Audemard É., Métois A., Theret L., Lisi V., Farah A., Spinella J.F., Chagraoui J., Moujaber O., Aubert L. (2024). Immunotherapeutic targeting of surfaceome heterogeneity in AML. Cell Rep..

[154] Pelosi E., Castelli G., Testa U. (2023). CD123 a Therapeutic Target for Acute Myeloid Leukemia and Blastic Plasmocytoid Dendritic Neoplasm. Int. J. Mol. Sci..

[155] Martino G., Cimino G., Caridi M., Perta G., Cardinali V., Sciabolacci S., Quintini M., Matteucci C., Venanzi A., Tiacci E. (2023). One disease, two faces: Clonally-related AML and MPDCP with skin involvement. Ann. Hematol..

[156] Jen E.Y., Gao X., Li L., Zhuang L., Simpson N.E., Aryal B., Wang R., Przepiorka D., Shen Y.L., Leong R. (2020). FDA Approval Summary: Tagraxofusp-erzs For Treatment of Blastic Plasmacytoid Dendritic Cell Neoplasm. Clin. Cancer Res..

[157] Zanotta S., Galati D., De Filippi R., Pinto A. (2024). Breakthrough in Blastic Plasmacytoid Dendritic Cell Neoplasm Cancer Therapy Owing to Precision Targeting of CD123. Int. J. Mol. Sci..

[158] Marra A., Akarca A.U., Martino G., Ramsay A., Ascani S., Perriello V.M., O’Nions J., Wilson A.J., Gupta R., Childerhouse A. (2023). CD47 expression in acute myeloid leukemia varies according to genotype. Haematologica.

[159] Narayan R., Piérola A.A., Donnellan W.B., Yordi A.M., Abdul-Hay M., Platzbecker U., Subklewe M., Kadia T.M., Alonso-Domínguez J.M., McCloskey J. (2024). First-in-human study of JNJ-67571244, a CD33 × CD3 bispecific antibody, in relapsed/refractory acute myeloid leukemia and myelodysplastic syndrome. Clin. Transl. Sci..

[160] Maslah N., Rety S., Bonnamy M., Aguinaga L., Huynh T., Parietti V., Giraudier S., Fenaux P., Cassinat B. (2024). Niclosamide combined to Azacitidine to target TP53-mutated MDS/AML cells. Leukemia.

[161] Mosna F., Borlenghi E., Litzow M., Byrd J.C., Papayannidis C., Tecchio C., Ferrara F., Marcucci G., Cairoli R., Morgan E.A. Long-term survival can be achieved in a significant fraction of older patients with core binding factor acute myeloid leukemia treated with intensive chemotherapy. Haematologica.

[162] Molica M., Perrone S., Mazzone C., Niscola P., Cesini L., Abruzzese E., de Fabritiis P. (2021). CD33 Expression and Gentuzumab Ozogamicin in Acute Myeloid Leukemia: Two Sides of the Same Coin. Cancers.

[163] Bernal T., Moreno A.F., de LaIglesia A., Benavente C., García-Noblejas A., Belmonte D.G., Riaza R., Salamero O., Foncillas M.A., Roldán A. (2023). Clinical outcomes after CPX-351 in patients with high-risk acute myeloid leukemia: A comparison with a matched cohort from the Spanish PETHEMA registry. Cancer Med..

[164] Niscola P., Tendas A., Mazzone C., Efficace F. (2019). Pain and related complaints in patients with acute leukemia: Time for simultaneous care in hemato-oncology. Support Care Cancer.

[165] El-Jawahri A., LeBlanc T.W., Kavanaugh A., Webb J.A., Jackson V.A., Campbell T.C., O’Connor N., Luger S.M., Gafford E., Gustin J. (2021). Effectiveness of Integrated Palliative and Oncology Care for Patients with Acute Myeloid Leukemia: A Randomized Clinical Trial. JAMA Oncol..

[166] Richardson D.R., Zhou X., Reeder-Hayes K., Jensen C.E., Islam J., Loh K.P., Gupta A., Basch E., Bennett A.V., Bridges J.F.P. (2024). Home Time Among Older Adults with Acute Myeloid Leukemia Following Chemotherapy. JAMA Oncol..

[167] Le R.Q., Przepiorka D., Chen H., Shen Y.L., Pulte E.D., Norsworthy K., Theoret M.R., De Claro R.A. (2024). Complete remission with partial hematological recovery as a palliative endpoint for treatment of acute myeloid leukemia. Blood.

[168] Geissler K., Koristek Z., Del Castillo T.B., Novák J., Rodríguez-Macías G., Metzelder S.K., Illes A., Mayer J., Arnan M., Keating M.M. Oral decitabine/cedazuridine versus intravenous decitabine for acute myeloid leukaemia: A randomised, crossover, registration, pharmacokinetics study. Br. J. Haematol..

[169] De Leeuw D.C., Ossenkoppele G.J., Janssen J.J.W.M. (2022). Older Patients with Acute Myeloid Leukemia Deserve Individualized Treatment. Curr. Oncol. Rep..

