# Correction: Niscola et al. Acute Myeloid Leukemia in Older Patients: From New Biological Insights to Targeted Therapies. *Curr. Oncol.* 2024, *31*, 6632–6658

**DOI:** 10.3390/curroncol32070386

**Published:** 2025-07-04

**Authors:** Pasquale Niscola, Valentina Gianfelici, Gianfranco Catalano, Marco Giovannini, Carla Mazzone, Nelida Ines Noguera, Paolo de Fabritiis

**Affiliations:** 1 Hematology Unit, S. Eugenio Hospital (ASL Roma 2), 00144 Rome, Italy; valentina.gianfelici@aslroma2.it (V.G.); marco.giovannini@aslroma2.it (M.G.); carla.mazzone@aslroma2.it (C.M.); paolo.de.fabritiis@uniroma2.it (P.d.F.); 2 Department of Biomedicine and Prevention, University of Rome Tor Vergata, 00133 Rome, Italy; gianfranco.catalano@uniroma2.it (G.C.); nelida.ines.noguera@uniroma2.it (N.I.N.); 3 Neurooncoemtology Units, Santa Lucia Foundation, I.R.C.C.S., 00143 Rome, Italy

In the original publication [1], there was a mistake in the legend for Figure 1. The caption incorrectly attributes the figure to reference [5] instead to reference [3], and uses the term “adapted”, which is not appropriate in this context and should be “reprinted”.

The correct legend appears below. The authors state that the scientific conclusions are unaffected. This correction was approved by the Academic Editor. The original publication has also been updated.

**Figure 1.** Effects of critical mutations on cellular function and pathophysiology of AML. In the cytoplasm, isocitrate is converted to alpha-ketoglutarate (A-KG). However, *IDH1* mutations reduce A-KG to D-2-hydroxyglutarate (D-2-HG), an oncometabolite. D-2-HG then travels to the nucleus and inhibits *TET2*, blocking DNA demethylation. Additionally, D-2-HG is created via reduction in the mitochondria by mutant *IDH2* enzymes from Krebs cycle-generated A-KG. *IDH1* inhibitors target the cytoplasmic reduction of A-KG to D-2-HG, while IDH2 inhibitors target the same process in the mitochondria. NPM1, which generally resides in the nucleolus and minimally binds XPO1, can travel to the nucleoplasm in stress conditions. In the nucleoplasm, it inhibits *HDM2*, which is significant because HDM2’s normal function is to inhibit *TP53*. Thus, by inhibiting *HDM2*, *NPM1* can increase *TP53*, which has important implications for cell regulation in stressful conditions. Mutant *NPM1* (NPM1c) has a higher affinity to *XPO1* and is prone to nuclear export, leading to critical protein export from the nucleus. Additionally, the consequent result of mutant NPM1 and *XPO1-NPM1c* can increase *HOX* expression. Furthermore, *NPM1c* and *KMT2Ar* interact with menin, facilitating leukemogenic cellular changes, which can be targeted via menin inhibition. Reprinted from Figure 1 in Ref. [3].

## References

[1] Niscola P., Gianfelici V., Catalano G., Giovannini M., Mazzone C., Noguera N.I., de Fabritiis P. (2024). Acute Myeloid Leukemia in Older Patients: From New Biological Insights to Targeted Therapies. Curr. Oncol..

